# High expression of CCDC6 in relation to unfavorable outcome and immune cells infiltration in hepatobiliary carcinoma

**DOI:** 10.7150/jca.76050

**Published:** 2022-09-21

**Authors:** Tianyu Wu, Xiaoqing Jiang, Bin Xu, Quan Zhong, Jinsheng Zheng, Xin Zhang, Yu Wang

**Affiliations:** 1 Department of Hepatobiliary Surgery, Nanfang Hospital, Southern Medical University, Guangzhou 510515, China.; 2 Surgical Intensive Care Unit, Nanfang Hospital, Southern Medical University, Guangzhou 510515, China.; 3 Department of Laboratory Medicine, Nanfang Hospital, Southern Medical University, Guangzhou 510515, China.

**Keywords:** CCDC6, hepatocellular carcinoma (HCC), Cholangiocarcinoma (CCA), Intrahepatic cholangiocarcinoma (iCCA), Histone acetylation, Immune infiltration

## Abstract

**Background:** The diagnosis of hepatobiliary carcinoma includes both hepatocellular carcinoma (HCC) and cholangiocarcinoma (CCA), the first and the second most common hepatobiliary malignancies, respectively. CCDC6 (coiled-coil domain-containing protein 6) is a protein that interacts with apoptosis and DNA damage response elements and is commonly detected in cells. The prognostic and biological roles of CCDC6 in hepatobiliary carcinoma remain unknown.

**Methods:** We used data from UALCAN, GEPIA, TIMER, GeneMANIA, STRING and HPA databases to determine the prognostic values and biological functions of CCDC6 in HCC and CCA. We downloaded the original online data from TCGA and GEO databases and analyzed them with R 3.2.2. We also gathered clinical records from patients with HCC (*n* = 94) and iCCA (*n* = 99) in our hospital to explore associations between CCDC6 expression and hepatobiliary carcinoma using immunohistochemistry detection. We used KEGG, GO and GESA analyses to explore relative pathways of CCDC6 in HCC and CCA. In addition, we assessed correlations between CCDC6 expression and tumor-infiltrating immune cells using data from the TIMER and GEPIA databases. Finally, we assessed associations between CCDC6 and marker genes of tumor-infiltrated immune cells in HCC to confirm some of our findings.

**Results:** The mRNA and protein expressions of CCDC6 were noticeably upregulated in HCC and CCA tissues as compared with the expressions in healthy control tissues. The high CCDC6 expression levels were significantly correlated with advanced tumor grades as well as poor prognosis in patients with HCC, but not in patients with CCA. Our functional enrichment analysis revealed that CCDC6 is mainly involved in cell cycle processes, gene transcription, and immune cell-related pathways. Moreover, we found that the CCDC6 levels were positively correlated with the presence of tumor-infiltrating immune cells, including macrophages, CD4+T cells and dendritic cells.

**Conclusion:** CCDC6 expression was increased in hepatobiliary carcinoma tissues. High expressions of CCDC6 were significantly associated with clinical severity variables (especially with advanced cancer stages and pathological tumor grades) and poor prognoses in patients with HCC. CCDC6 upregulation is associated with histone acetylation and immune infiltration in hepatobiliary carcinoma. In addition, CCDC6 has the potential to be used as a predictive biomarker during targeting therapy and immunotherapy.

## Introduction

Primary liver and biliary tract tumors can be divided into intrahepatic and extrahepatic types. Hepatocellular carcinoma (HCC) and intrahepatic cholangiocarcinoma (iCCA) are the most common primary intrahepatic malignancies, encompassing 80-90% and 10-20% of cases, respectively; while the extrahepatic malignancies include perihilar and distal cholangiocarcinomas 1-3. Diagnosis of HCC and iCCA has traditionally been done on the basis of radiologic, serologic and/or pathologic findings. During early-stage HCC (stage 0/A, according to the Barcelona Clinic Liver Cancer [BCLC] staging system), the most effective therapeutic options are surgical resection, liver transplantation, or percutaneous local ablation 4. During this early stage, the median OS is >60 months with a 5-year survival of 60-80%, but a 5-year recurrence of 70%. However, most HCCs are diagnosed at an intermediate (stage B) or an advanced stage (stage C), when the median OS is only approximately 11-20 months with a 5-year survival of 16% 5. What's worse, the 5‐year survival rates range from 2% to 15% for iCCA 6. Despite advances in treatment options such as surgery, chemotherapy, radiotherapy, immunotherapy, and targeted therapy, the high mortality rate of this disease remains a global challenge 7,8. Therefore, identifying specific biomarkers and therapeutic targets to distinguish HCC and CCA and their molecular mechanisms is important.

CCDC6 (coiled-coil domain-containing protein 6) is a tumor suppressor gene in human chromosome 10q2l, its product is involved in apoptosis and the DNA damage response. It was originally detected while studying recombinant genes caused by chromosomal translocation involving the RET proto-oncogene in some thyroid tumors 9. In primary tumors, an abnormal CCDC6 function could influence genome stability and contribute to carcinogenesis 10. The CCDC6 product is an extensively expressed 65 kDa nuclear and cytosolic protein, phosphorylated by an extracellular signal-regulated protein kinase following serum stimulation 11. We have reported a patient-derived iCCA xenograft mouse model endogenously expressing an FGFR2-CCDC6 fusion protein and produced preliminary evidence for the role of CCDC6 in tumor promotion 12. Interestingly, we found associations between CCDC6 and hepatobiliary tumors using integrative bioinformatics analysis tools as well as immunohistochemical (IHC) detection. Therefore, we further investigated the distinctive genomic alterations and functional networks associated with CCDC6 expression and evaluated its role in tumor targeted therapy and immunotherapy. Our findings suggest that CCDC6 expression may be useful as a prognostic biomarker during targeted therapy and immunotherapy, and they provide insights into the molecular mechanisms that differ between patients with HCC and those with CCA.

## Materials and Methods

### Bioinformatics analysis

#### TIMER/TIMER 2.0 database analysis

We looked at data in the TIMER/TIMER 2.0 database to explore the CCDC6 expression profiles and the abundances of immune infiltrates in both HCC and CCA tissues. We also applied TIMER 2.0, another database using a statistical deconvolution method, to deduce the abundance of tumor-infiltrating immune cells from gene expression profiles (association between CCDC6 expression and presence of immune cells in HCC and CCA) 13. Gene expression levels are represented as log_2_ TPM values.

#### UALCAN database analysis

We evaluated the different expressions of CCDC6 between 33 kinds of cancers and their corresponding normal tissues in the UALCAN database 14. Moreover, we included patients' clinical data to perform our analysis. We applied Student's t-tests to assess the significance of differences and considered those with *p* < 0.05 as statistically significant.

#### GEPIA 2.0 database analysis

GEPIA 2.0 is an online database that facilitates the standardized analysis of RNA-seq data from 9,736 cancer samples and 8,587 normal control samples in the TCGA and GTEx data sets 15. Therefore, we used this database to evaluate the association between CCDC6 expression and patients' prognoses for multiple cancer types, and we plotted overall survival (OS) and recurrence-free survival (RFS) curves among them. In addition, we also examined the associations between CCDC6 expression and the prognosis of patients, including the disease-specific survival (DSS), disease-free interval (DFI), and progression-free interval (PFI) in HCC and CCA using the TCGA database with the help of R software.

#### GO, KEGG, GSEA and GSVA analysis

We performed Gene Ontology enrichment analysis for biological processes (BP), cellular components (CC), and molecular functions (MF), and KEGG pathway analysis for all the differentially expressed genes (DEGs) shared in the stromal and immune groups. GO analyses allowed us to examine the biological and molecular functions of CCDC6 in HCC and CCA tissues. We also used GSEA and GSVA to determine the potential molecular mechanisms of CCDC6 in the same tissues. All the analyses were conducted using the R package ClusterProfiler.

#### Human Protein Atlas (HPA) database analysis

We retrieved the protein expression profiles of CCDC6 in HCC, iCCA and corresponding normal tissues from the HPA. HPA is a website designed to map all human proteins in cells, tissues and organs by integrating various omics technologies (including antibody-based imaging, mass spectrometry-based proteomics, transcriptomics and systems biology) 16. We used the HPA database to analyze the protein expressions of CCDC6 in normal liver tissues, HCC and iCCA tissues. In addition, we performed immunohistochemical (IHC) analyses.

#### Data and Software Availability

We obtained all original online data of HCC and CCA for analysis from The Cancer Genome Atlas (TCGA) and Gene Expression Omnibus (GEO) databases. R 3.2.3 was used to integrate the original data and verify the results analyzed in the website database.

### Patient cohort and ethical approval

Patients underwent tumor resections in Nanfang Hospital (Southern Medical University), we analyzed those that were pathologically confirmed as HCC or iCCA from 2007 to 2016. We enrolled 94 patients with HCC and 99 with iCCA in our study and collected individual gender, age, pathological grade, clinical stage, treatment, and other clinical follow-up data. We also retrospectively collected the corresponding formalin-fixed, paraffin-embedded tissues in our institutional biobank. The Nanfang Hospital of Southern Medical University Biomedical Research Ethics Committee approved this research, which was conducted following the ethical guidelines required in the Declaration of Helsinki (Official Number. NFEC-2022-056). Case inclusion criteria: The patient was diagnosed with liver cancer for the first time and underwent surgical resection. The postoperative pathological results suggested HCC or iCCA. At the same time, the patient had no other tumors or serious fatal diseases, and both of the clinical data and follow-up information of the patient were complete. Case exclusion criteria: Other pathological types of liver cancer (Extrahepatic cholangiocarcinoma, mixed hepatocellular carcinoma, liver sarcoma, etc.) and liver metastasis were excluded. Patients with other tumors or serious fatal diseases were also removed. Incomplete medical history data was also removed.

### Immunohistochemistry analysis

We performed IHC staining following a standard automation protocol with a rabbit polyclonal antibody against human CCDC6 (Abcam). Briefly, after dewaxing and hydration, antigen retrieval was achieved in a citrate buffer (pH 6.0). The sections were sealed with 10% normal goat serum for 30 minutes at room temperature, after adding the first antibody (1% BSA) the samples were incubated overnight at 4 °C. The first antibody was detected using a biotinylated secondary antibody with the help of an HRP conjugated SP system. Three pathologists examined CCDC6 immunostaining in HCC and iCCA samples. At least two pathologists discussed and reviewed difficult samples to reach a consensus. Immunoreactivity scores were calculated by multiplying the number representing the percentage of immunoreactive cells (**1** for percentages < 1%; **2** for percentages between 1 and 10%; **3** for percentages between 11 and 50%; and **4** for percentages >50%) by the number representing the dyeing intensity (0 for absence of dye; 1 for weak dye detection; 2 for moderate detection; and 3 for strong detection). We classified the CCDC6 expression scores as negative (**0-2**), mildly positive (**3-4**), moderately positive (**5-8**), or highly positive (**9-12**).

### Statistical analysis

We applied Chi-square and Fisher exact tests to compare differences in pathological and molecular characteristics among the different patient groups. Cox regression analysis and the Kaplan-Meier method were used to evaluate the prognostic factors. We generated a Kaplan-Meier curve to calculate survival rates and compared them using a logarithmic rank test. The significance of prognostic factors was evaluated by univariate and multivariate Cox proportional risk regression, and we considered *p* values > 0.05 as statistically significant. All analyses were performed with the SPSS 24 and GraphPad 8.0. T softwares.

## Results

### CCDC6 was overexpressed in patients with either HCC or CCA

**Figure 1:**
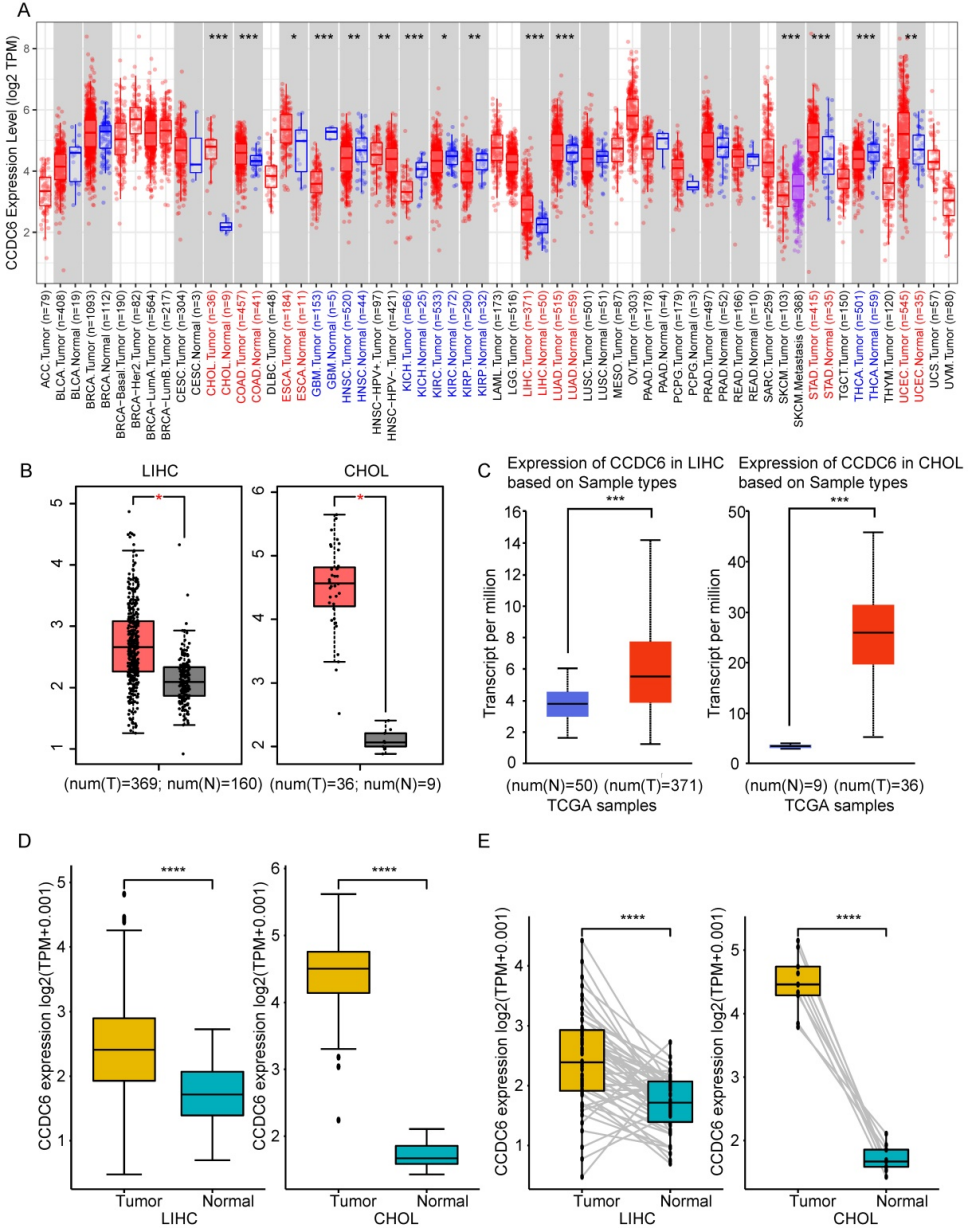
** CCDC6 expression was high in patients with either HCC or CCA. (A)** The CCDC6 expression of 39 cancers (including specific cancer subtypes) was analyzed with TIMER2. **(B)** Higher expression of CCDC6 in HCC and CCA compared to normal tissues in the GEPIA database. **(C)** Higher expression of CCDC6 in HCC and CCA compared to normal tissues using UALCAN database. **(D)** Confirmation of CCDC6 expression in tumor and adjacent normal tissues in the TCGA database. **(E)** Confirmation of CCDC6 expression of paired samples in TCGA database. * P < 0.05; ** P < 0.01; *** P < 0.001.

We applied the TIMER2 approach to analyze the expression profiles of CCDC6 across various cancer types in the TCGA database. The CCDC6 expression levels in the tumor tissues of cholangiocarcinoma (*p*<0.001), colon adenocarcinoma (*p<*0.001), esophageal carcinoma (*p*<0.05), liver hepatocellular carcinoma (*p*<0.001), lung adenocarcinoma (*p*<0.001), stomach adenocarcinoma (*p*<0.001), and uterine corpus endometrial carcinoma (*p*<0.01) are higher than those in the corresponding control tissues, as shown in **Figure 1A**. By contrast, the CCDC6 expression levels in glioblastoma multiforme (*p*<0.001), head and neck squamous cell carcinoma (*p*<0.01), kidney chromophobe (*p*<0.001), kidney renal clear cell carcinoma (*p*<0.05), kidney renal papillary cell carcinoma (*p*<0.01) and thyroid carcinoma (*p*<0.001) were lower than those in the corresponding control tissues. The CCDC6 expression levels in skin cutaneous melanoma are also lower than those in the corresponding metastatic lesions (*p*<0.001). We further evaluated the CCDC6 expression difference between the normal tissues and tumor tissues of cholangiocarcinoma and liver hepatocellular carcinoma using GEPIA (**Figure 1B**) and UALCAN (**Figure 1C**), and we found that both tumor tissues overexpressed CCDC6. The paired analysis results from the TCGA database (**Figure 1D and E**) further confirmed that CCDC6 is highly expressed in cholangiocarcinoma and liver hepatocellular carcinoma (all *p*<0.001) compared with the expression in the corresponding normal controls, the difference was highest in the cholangiocarcinoma.

These findings suggest that CCDC6 expression is increased in both patients with HCC and those with CCA.

### The CCDC6 expression was closely correlated with clinical variables and the prognosis of patients with HCC and CCA, especially in the cases of HCC

**Figure 2:**
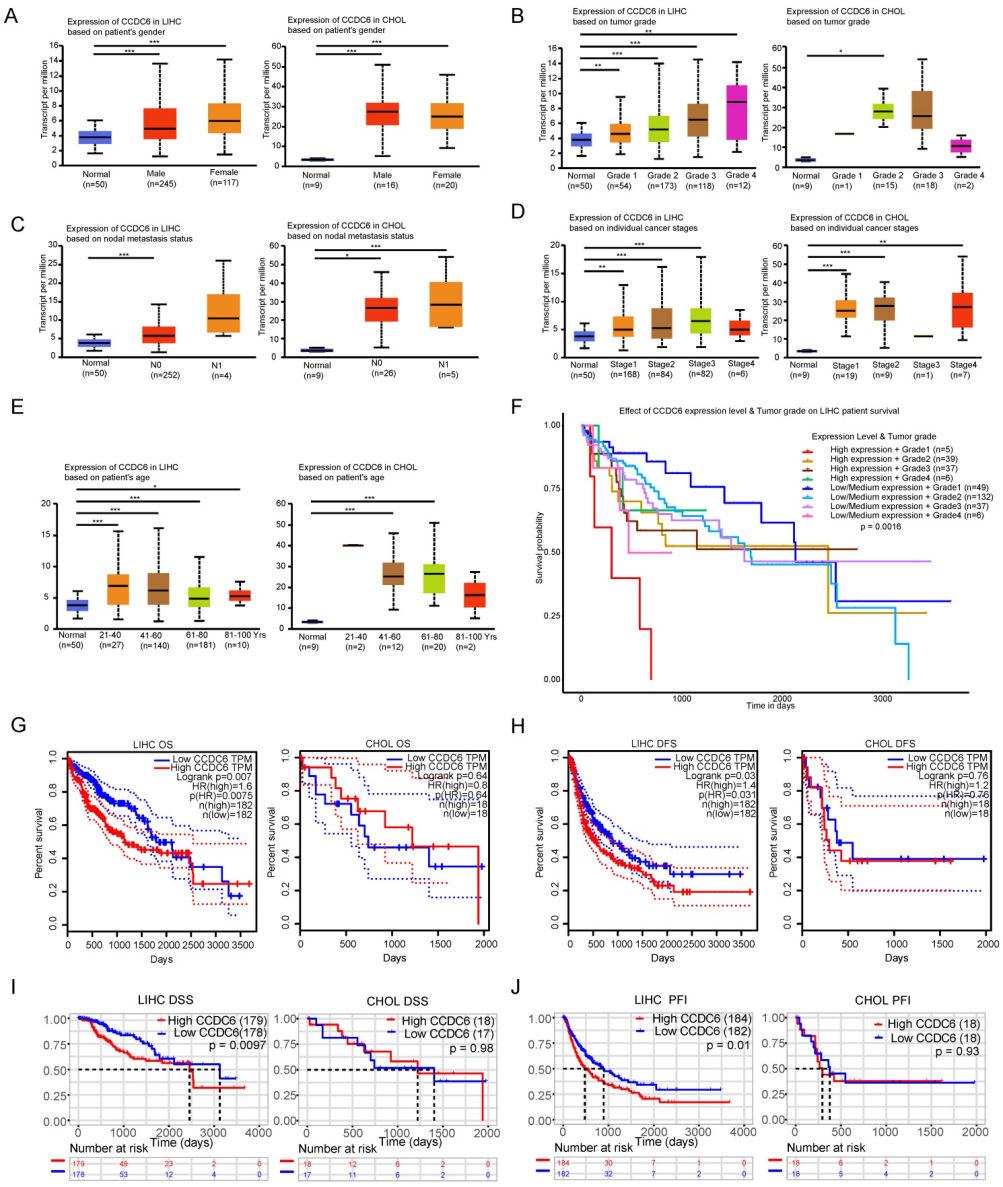
The CCDC6 expression level in tumors was closely correlated with clinical variables and the prognosis of patients with HCC and CCA, especially in the case of HCC. Analysis is shown for gender **(A)**, tumor grade** (B)**, nodal metastasis status **(C)**, individual cancer stages **(D)** and ages **(E)**. Higher levels of tumor grades were associated with poor prognosis in HCC patients of UALCAN database **(F)**. Overexpression levels of CCDC6 were associated with shorter OS, DFS, PFI, DSS, DFI in HCC from GEPIA **(G-H)** and acquired TCGA database **(I-J)**. No significant associations were found between CCDC6 and CCA from GEPIA (G-H) and acquired TCGA database (I-J). *p < 0.05, **p < 0.01, ***p < 0.001.

We applied UALCAN to investigate CCDC6 expressions among groups of patients according to different clinical variables. CCDC6 expression was significantly upregulated in both men and women with HCC/CCA as compared to the levels in the corresponding normal groups (**Figure 2A**). We found a gradual significant increase in the CCDC6 expression of patients with HCC according to their tumor grade (from 1, well differentiated; to 2, moderately differentiated; to 3, poorly differentiated; and to 4, undifferentiated) and in patients with in stage 2 CCA (**Figure 2B**). On the basis of the nodal metastasis status, the CCDC6 expression was higher in patients with HCC classified as N0 (No regional lymph node metastasis) and in those with CCA classified as N0 or N1 (metastases in 1 to 3 axillary lymph nodes;** Figure 2C**). We saw a clear increase in CCDC6 expression in patients with stage 1, 2 or 3 HCC and in patients with stage 1, 2 or 4 CCA (**Figure 2D**). The CCDC6 level was also significantly elevated in patients with HCC from different age groups (21-40 years, 41-60 years, 61-80 years and 81-100 years) and in patients with CCA (41-60 years and 61-80 years; **Figure 2E**). Moreover, we found CCDC6 expression upregulation in HCC patients with TP53 mutation or wild-type TP53 as compared to the expression in normal control patients (Supplementary Figure 1A). Above all, these results reveal a close correlation between CCDC6 expression and clinical variables, especially for HCC.

We observed an interesting phenomenon in the association between CCDC6 and the prognosis of patients with HCC, the survival of patients with CCDC6 positive expression got worse with higher tumor grades in the UALCAN analysis (**Figure 2F**). Thus, we evaluated the association between CCDC6 expression and HCC/CCA prognosis using the GEPIA database. The OS curves according to the CCDC6 expression levels are displayed in **Figure 2G**. Notably, high transcriptional levels of CCDC6 (*p=*0.0075) were markedly associated with shorter OS in patients with HCC. High transcriptional levels of CCDC6 (*p*=0.031) were remarkably associated with shorter DFSs in patients with HCC (**Figure 2H**), and we found similar results in terms of PFIs (*p*=0.01), DSSs (*p*=0.0097), and DFIs (*p=*0.083) calculated from TCGA data (**Figure 2I, J and Supplementary Figure 1B**). By contrast, we found no association between CCDC6 levels and CCA from GEPIA or acquired TCGA data: OS (*p*=0.64), DFS (*p*=0.76), DSS (*p*=0.98), PFI (*p*=0.93), DFI (*p*=0.15) (**Figure 2I, J and Supplementary Figure 1C**). The expression levels of CCDC6 increased with HCC progression. These findings indicate that the CCDC6 level is closely correlated with clinical variables and poor prognoses in HCC and CCA, especially in the case of HCC.

### Confirmation of CCDC6's association with poor prognosis of patients with HCC and its distribution in tumor cells using IHC detection

**Figure 3:**
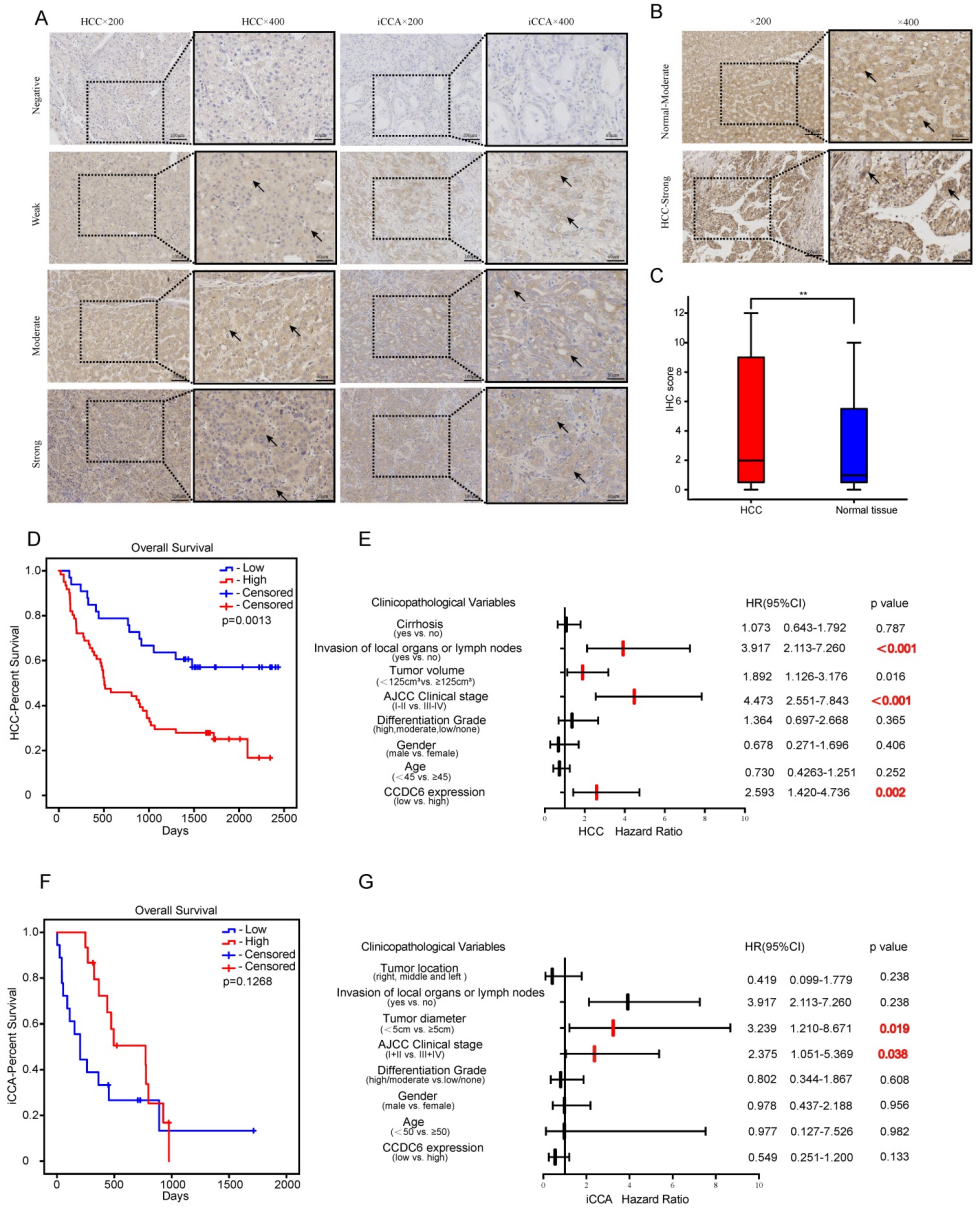
CCDC6's distribution in tumor cells using Immunohistochemical (IHC) detection and the confirmation of its relation with poor prognosis of HCC patients. Representative IHC-stained of HCC and iCCA **(A)** (negative, weak, moderate, strong). **(B-C)** Typical IHC-stained of paired tissue and the corresponding statistical analysis of HCC. **(D)** Overexpression levels of CCDC6 were associated with shorter OS of HCC in our groups. **(F)** No significant association were found between CCDC6 and OS of CCA in our groups. **(E, G)** Forest plots of HCC/iCCA displaying hazard ratios (HRs) and 95% confidence intervals for the clinical parameters. * P < 0.05; ** P < 0.01; *** P < 0.001.

To further investigate CCDC6 expression in hepatobiliary cancers, we performed IHC analyses of 94 paraffin-embedded HCC tissues including both carcinoma tissues and their matched adjacent non-carcinoma tissues. Intrahepatic cholangiocarcinoma shares more similar clinical and histopathologic features with hepatocellular carcinoma than with extrahepatic cholangiocarcinoma 17. Therefore, we analyzed another 99 paraffin-embedded iCCA tissues with clinical data. CCDC6 was mostly expressed in the cytoplasm and partly in the nucleus, as showed in our photo of HCC and iCCA tissues. **Figure 3A** shows representative IHC-stained slides displaying the dye intensities and the CCDC6 expressions (negative, weak, moderate and strong) of HCC/CCA tissues. IHC results also indicate that the tumor tissues expressed significantly higher CCDC6 protein than the matched adjacent non-carcinoma tissues. **Figure 3B-C** shows representative IHC-stained slides displaying with their corresponding CCDC6 expressions quantifications in HCC. In addition, the online IHC data of HCC/iCCA from the Human Protein Atlas (HPA) and representative IHC-stained slides are displayed in **Supplementary Figure 2** (A-B HCC; C iCCA; D normal liver tissue).

On the basis of our results, we also explored the potential correlation between CCDC6 protein expression and the clinicopathological features of patients with HCC/iCCA. The association between clinical variables of patients with HCC and the level of CCDC6 expression is described in **Table 1**: 64.9% (61/94) of the patients with HCC exhibited high CCDC6 expression, while 35.1% (33/94) of them showed relatively low CCDC6 expression. The examined samples belonged to patients with clinical stage I-II in 52.1% (49), and clinical stage III-IV in 47.9% (45). These results show that the expression levels of CCDC6 were significantly correlated with multiple variables, including differentiation grade (*p*=0.005), AJCC clinical stage (*p*=0.038), and death (*p*=0.001); but not with age (*p*=0.933), gender (*p* = 0.732), tumor volume (*p*=0.829), Invasion of local organs or lymph nodes (*p*=0.054) or the presence of cirrhosis (*p*=0.077). **Table 2** displays the association between the clinical variables of patients with iCCA and their CCDC6 expression levels: 52.5% (52/99) of the patients exhibited high CCDC6 expression, while 47.5% (47/99) of them showed relatively low CCDC6 expression levels. Among all the clinical variables, age, gender, tumor diameter, differentiation grade, invasion of local organs or lymph nodes, AJCC clinical stage, tumor location, and death were not significantly correlated with CCDC6 expression.

We evaluated the potential for CCDC6 levels to predict OSs in patients with HCC/iCCA by comparing the OSs of patients with high CCDC6 expression to those with low CCDC6 expression. As for the 94 patients with HCC in our group, those with higher CCDC6 expression tended to have shorter OSs (*p* = 0.013; **Figure 3D**). A cohort of 33 patients with iCCA (only 33 from 99 with complete survival data) revealed a lack of association between the OS and the CCDC6 expression levels (**Figure 3F**). We conducted univariate and multivariate Cox regression analyses to further investigate the independent prognostic value of CCDC6 expression levels. The univariate Cox regression analysis revealed that CCDC6 expression, AJCC clinical stage, tumor volume, invasion of local organs or lymph nodes and clinical state are associated with the OS of patients with HCC (**Table 3**). Further, the multivariate Cox regression analysis showed that CCDC6 expression and the clinical state were correlated with poor OS in patients with HCC (**Table 3**). As for patients with iCCA, the multivariate Cox regression analysis showed that CCDC6 expression, poor clinical state, and a large tumor diameter were correlated with a poor overall survival (**Table 4**). We also drew Forest plots to display hazard ratios (HRs) and 95% confidence intervals for the clinical variables and OS in patients with HCC/iCCA (**Figure 3E and G**).

Overall, our investigations confirmed that CCDC6 expression (mostly distributed in the cytoplasm according to our IHC detection) is associated with poor prognoses in patients with HCC.

### Identification of CCDC6-interacting genes and proteins

**Figure 4:**
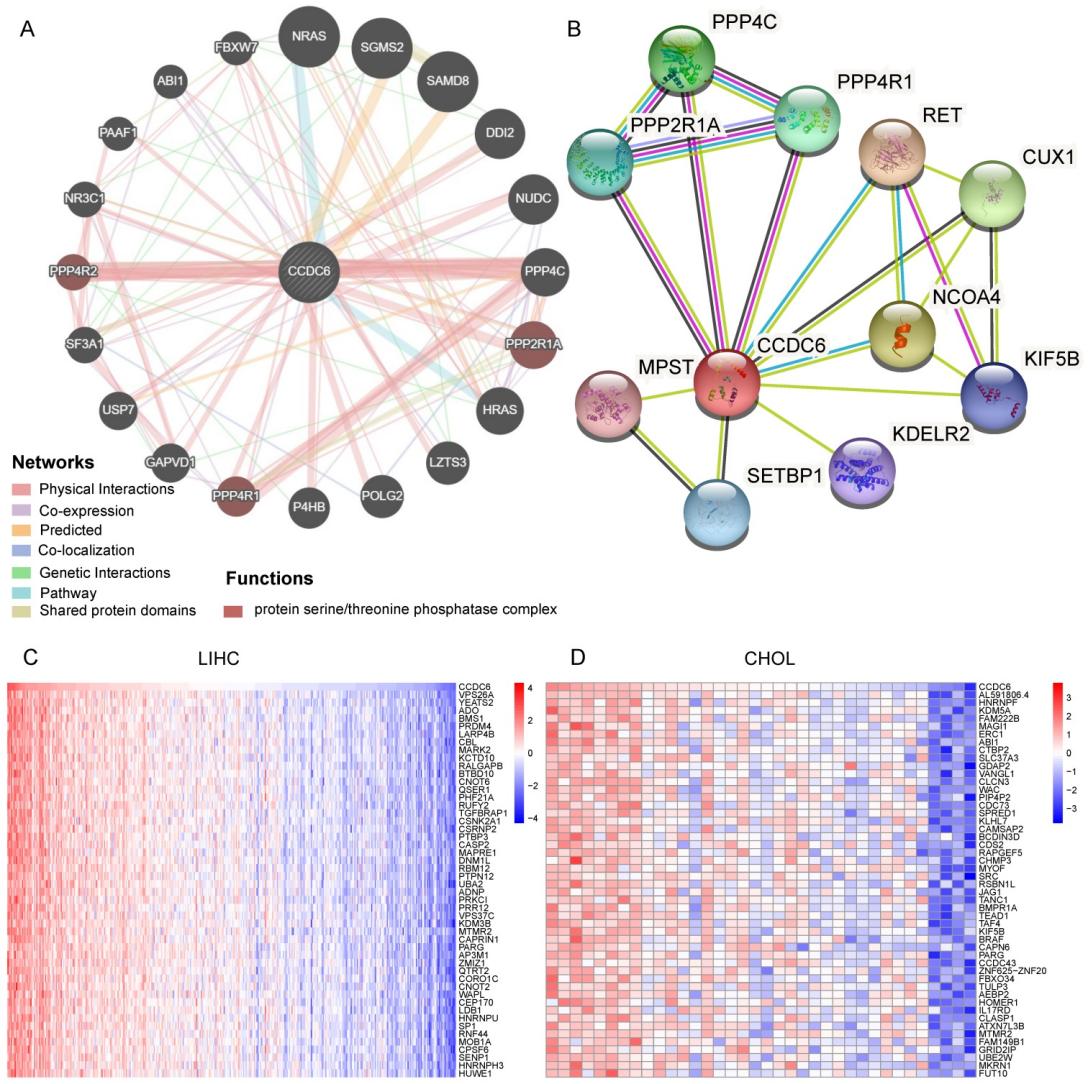
Identification of CCDC6-Interacting Genes and Proteins. **(A)** The 20 most frequently altered genes closely correlating with CCDC6 from GeneMania. **(B)** The 10 most frequently proteins closely correlating with CCDC6 from STRING database. **(C)** The top 50 positive genes in HCC and CCA from TCGA database.

We used GeneMania to assess gene-gene interactions for CCDC6 and other DEGs 18. The main 20 DEGs correlating with CCDC6 are shown in **Figure 4A** and include NRAS, NUDC, PPP4C, PPP2R1A, USP7, and NR3C1. Our functional analysis results imply that these genes belong to the protein serine/threonine phosphatase complex, which plays a key role in numerous cellular mechanisms including cell proliferation, cell migration, regulation of cell death/survival balance, inflammation and autoimmunity. We also used the STRING online website to investigate the protein-protein interaction (PPI) network of CCDC6 19. We found 20 edges and 11 nodes, including PPP4C, PPP4R1, PPP2R1A, and CUX1 (**Figure 4B**). Interestingly, PPP4C and PPP2R1A are negative regulators of HDAC3 (Histone Deacetylase 3) activity, and inhibition of HDAC3 blocks the induction of PD-L1 expression 20,21. Knockdown of the deubiquitinase USP7 in functional regulatory T (Treg) cells also abrogates their ability to resolve inflammation both *in vitro* and *in vivo* 22,23. Otherwise, PPP2R1A may be involved in the regulation of T cell functions in autoimmunity diseases 24,25. Next, we used the TCGA database to identify positive or negative genes co-expressed with CCDC6. The top 50 positive or negative genes in HCC and CCA are showed in **Figure 4C-D and Supplementary Figure 3A-B**.

The above analysis revealed related genes as well as their products, those most closely related to CCDC6 include PPP4C, PPP4R1, PPP2R1A and others.

### GO and KEGG Pathway Analysis results for CCDC6 in HCC and CCA

**Figure 5:**
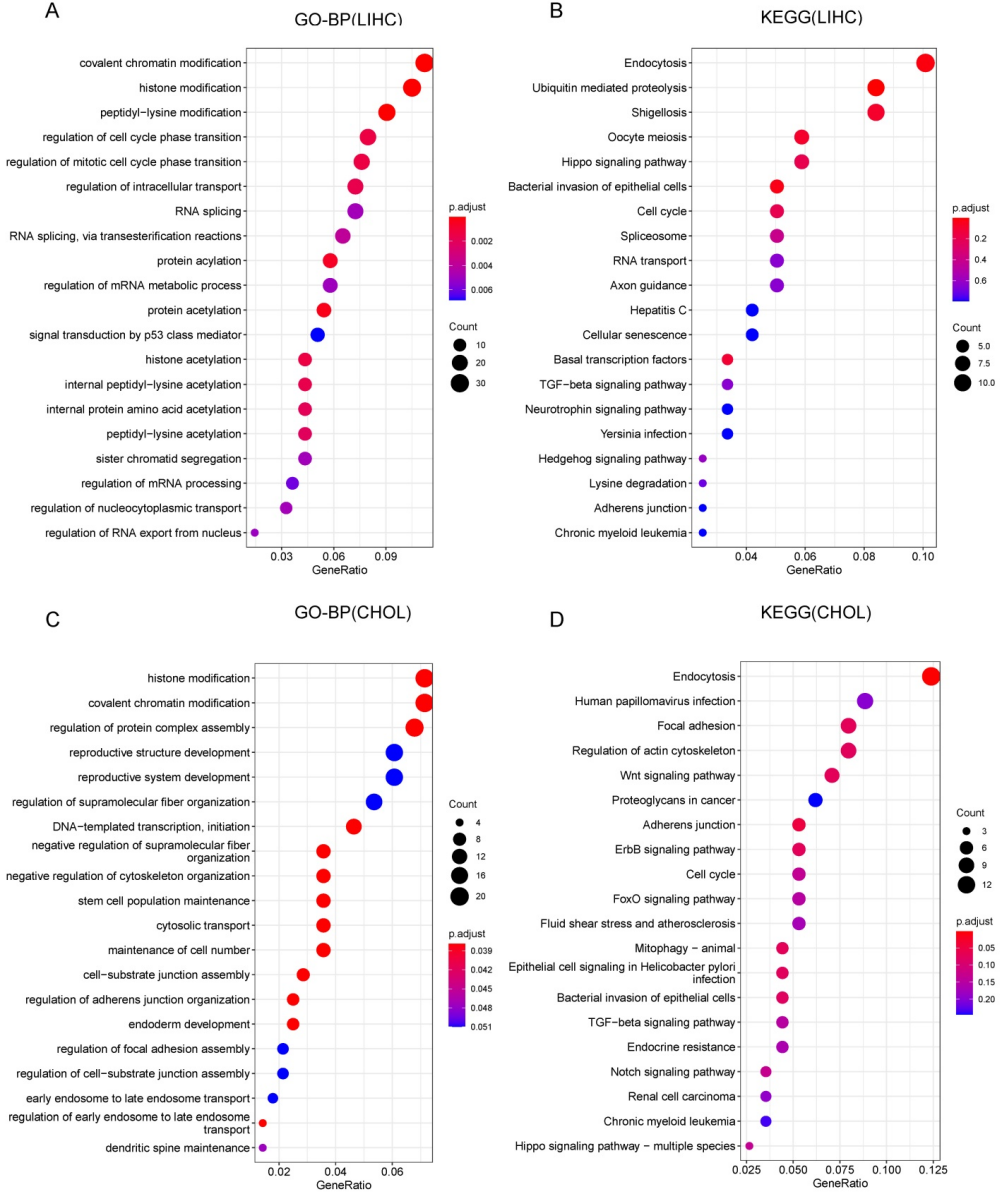
Gene Ontology (GO) and Kyoto Encyclopedia of Genes and Genomes (KEGG) Pathway Analysis results for CCDC6 in HCC and CCA from TCGA database. **(A)** Top 20 enrichment terms in BP categories in HCC. **(B)** Top 20 KEGG enrichment pathways in HCC. **(C)** Top 20 enrichment terms in BP categories in CCA. **(D)** Top 20 KEGG enrichment pathways in CCA.

To further investigate the role of CCDC6 in HCC and CCA, we conducted GO (Gene Ontology) and KEGG (Kyoto Encyclopedia of Genes and Genomes) enrichment analyses to reveal possible CCDC6-relative pathways and biological functions. The top 20 significant terms of CC, MF and BP enrichment analyses are presented in **Supplementary Figure 4 and Figure 5**. Among the CC, MF and BP of HCC, the significant terms were histone acetyltransferase complex, ubiquitin-like protein transferase activity, histone acetyltransferase activity, covalent chromatin modification, and regulation of cell cycle phase transition (**Supplementary Figure 4A-B, Figure 5A**). The HCC KEGG data revealed the top 5 most enriched terms (endocytosis, ubiquitin mediated proteolysis, shigellosis, Oocyte meiosis, and Hippo signaling pathway; **Figure 5B**). As for CCA, the frequent terms in CC, MF and BP were histone acetyltransferase complex, cell adhesion molecule binding, histone modification, regulation of protein complex assembly, and reproductive structure development (**Supplementary Figure 4C-D, Figure 5C**). The top 5 most enriched terms in the KEGG analysis of CCA were endocytosis, human papillomavirus infection, focal adhesion, regulation of actin cytoskeleton, and the Wnt signaling pathway (this last term correlates with immune escape through defective recruitment of dendritic cells; **Figure 5D**). These results revealed the role of CCDC6 in cell cycle processes, gene transcription, and DNA damage repair, especially the function related with histone acetylation in HCC.

### GSEA results identified CCDC6-related signaling pathways

**Figure 6:**
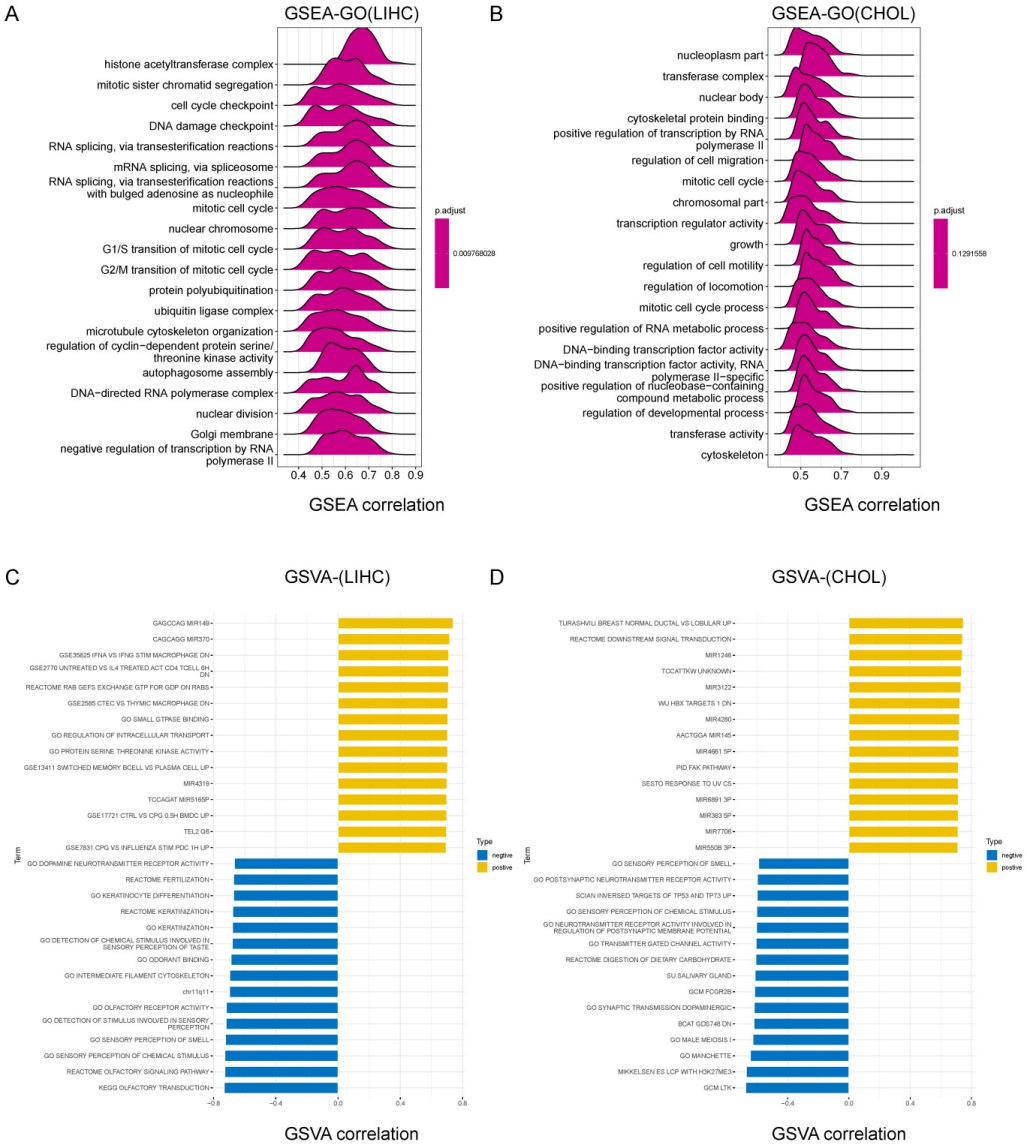
Gene Set Enrichment Analysis (GSEA) results identified CCDC6-related signaling pathways. Top 20 enrichment terms in GO **(A)** category in HCC. Top 20 enrichment terms in GO **(B)** in CCA. Top 15 positive/negative enrichment terms in GSVA **(C)** category in HCC. Top 15 positive/negative enrichment terms in GSVA **(D)** category in HCC.

We further explored the molecular mechanisms affected by CCDC6 in HCC/CCA by conducting a GSEA (Gene Set Enrichment Analysis). Among the GO, KEGG and Reactome result data of GSEA in HCC, the frequent signaling pathways influenced by CCDC6 were enriched in the cell cycle process and gene transcription terms including histone acetyltransferase complex, mitotic sister chromatid segregation, cell cycle checkpoint, Ubiquitin-mediated proteolysis, and adherens junctions. We also found important pathways related to HCC, such as DNA damage checkpoint, T cell receptor signaling pathway, ErbB signaling pathway, Notch signaling pathway, VEGFA-VEGFR2 pathway, programmed cell death, tyrosine kinases receptor signaling, and adaptive immune system (**Figure 6A, Supplementary Figure 6A-B**). For CCA, we obtained similar terms displayed, including mitotic cell cycle and regulation of cell migration (**Figure 6B, S6C-D**). We decided to use the GSVA to further explore the biological processes involving CCDC6 in HCC/CCA. The top 15 pathways with positive or negative correlations with CCDC6 expression are listed as below (**Figure 6C-D**). CCDC6 expression in HCC is positively correlated with several immune cells (including macrophages, CD4+T cells and memory B cells) and the regulation of transcription and translation processes. In contrast, the expression of CCDC6 in HCC is negatively correlated with the olfactory signaling pathway, sensory perception of chemical stimuli and sensory perception of smell.

Our GSEA and GSVA results on the TCGA database analysis indicate that CCDC6 may be related to histone acetylation and the infiltrations of tumor immune cells in HCC.

### Correlation analysis between CCDC6 expression and infiltrating immune cells

**Figure 7:**
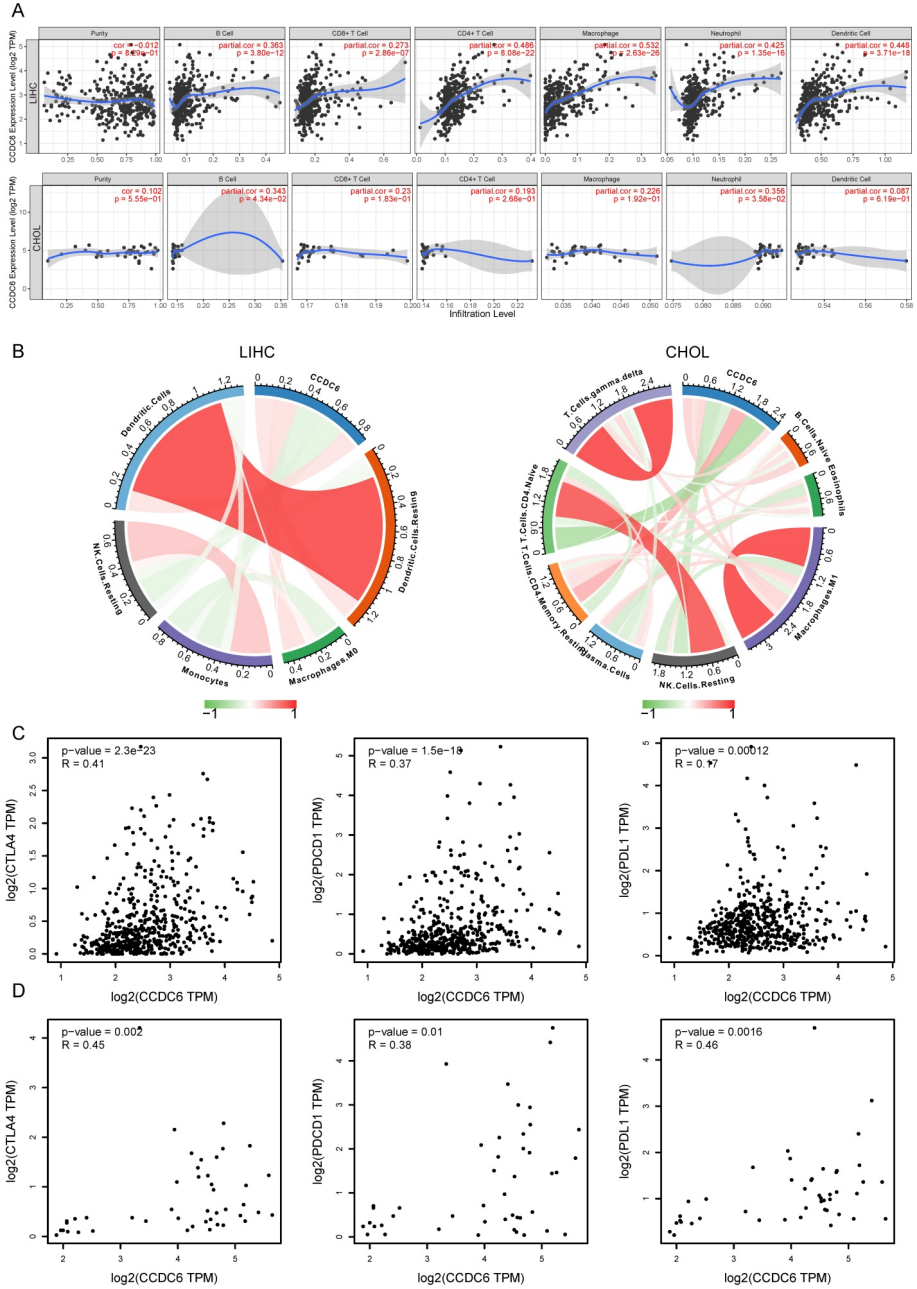
Correlation of CCDC6 expression with immune infiltration cells. **(A)** CCDC6 is positively correlated with the infiltration of different immune cells in HCC using the TIMER database. **(B)** CCDC6 expression is relative to the infiltration of immune cells in HCC using the CIBERSORT algorithm. **(C, D)** Scatterplots of the correlations between CCDC6 expression and PD-1, PD-L1 and CTLA-4 in HCC and CHOL using the GEPIA database.

Tumor infiltrating lymphocytes affect the patients' survival in various tumors. Here, we first investigated the correlations between CCDC6 expression and six types of infiltrating immune cells including B cells, CD8+ T cells, CD4+ T cells, macrophages, neutrophils, and dendritic cells using TIMER. All types of infiltrating immune cells had a significant positive correlation with CCDC6 expression levels in patients with HCC (**Figure 7A**). In contrast, only B cells and neutrophils had a low positive correlation with CCDC6 expression levels in patients with CCA (**Figure 7A**). To further evaluate the effect of CCDC6 expression on the tumor microenvironment, we investigated these correlations using the established computational resource CIBERSORT. Significantly, CCDC6 was positively correlated with the infiltration levels of dendritic cells, resting dendritic cells, M0-macrophages, and resting CD4-memory T cells; but negatively correlated with the infiltration levels of mast cells, resting mast cells, monocytes, resting NK Cells, and naive CD4 T Cells in HCC (**Figure 7B, Supplementary Figure 5A**). In CCA tumors, CCDC6 was only positively correlated with the infiltration levels of resting T cells CD4-Memory, but negatively correlated with the infiltration levels of resting NK cells and naïve CD4 T cells (**Figure 7B, Supplementary Figure 5B**). We further investigated the interrelationship between CCDC6 expression and typical T cell checkpoints, such as CTLA-4, PDCD1 and PD-L1 in the GEPIA database. CCDC6 expression was significantly correlated with the expression of PD-1, PD-L1 and CTLA-4 in HCC but not in CCA (**Figure 7C-D**).

These findings further support the hypothesis that CCDC6 expression is significantly associated with immune infiltration and suggest that CCDC6 has an important role in immune escape in HCC microenvironments.

### Correlation analysis between CCDC6 expression and related markers of immune cells using TIMER database

We investigated the correlations between CCDC6 expression and diverse immune markers in both HCC and CCA tumors using the TIMER database. The genes listed in **Table 5** were used to characterize immune cells, including B cells, T cells, CD8+ T cells, monocytes, tumor-associating macrophages (TAMs), M1 macrophages, M2 macrophages, neutrophils, NK cells and dendritic cells. Tumor purity affects the assessment of immune infiltration in clinical cancer biopsies. After adjusting for tumor purity, we found that the CCDC6 expression was significantly associated with most immune markers in divergent types of immune cells in HCCs, while most markers of immune cells were not associated with CCDC6 expression in CCAs (**Table 5**).

These results suggest that CCDC6 is significantly associated with most immune markers in divergent types of immune cells in HCC.

## Discussion

### The overexpression of CCDC6 is associated with poor HCC prognosis

An estimate by the European Association for the Study of the Liver (EASL) in their guidelines for the management of hepatocellular carcinoma (HCC) states that more than 1 million people will die due to liver cancer worldwide in 2030 4. HCC and iCCA, the most common cancer types in hepatobiliary carcinoma, are often diagnosed at an advanced stage and present poor prognoses. Thus, mechanisms inducing hepatobiliary carcinoma metastasis and significant prognostic biomarkers of hepatobiliary carcinoma need to be identified. In our study, by means of bioinformatics analyses of the TIMER, GEPIA, UALCAN and TCGA public databases we showed that the expression of CCDC6 in hepatobiliary carcinoma was higher than that in normal liver tissues (**Figure 1**). Subsequently, we investigated the clinical prognostic significance of CCDC6 in hepatobiliary carcinoma. High expression of HCC was significantly correlated with sex, age, clinical stage, histological grade, and the presence of metastasis in patients with HCC (**Figure 2**). Moreover, survival analyses from GEPIA, UALCAN and TCGA public databases indicate that patients with HCC and high CCDC6 expression exhibit a markedly worse survival rate than those with low CCDC6 expression (**Figure 2**). However, we found no significant correlations between CCDC6 expression and CCA. These results indicate that CCDC6 may be an independent prognostic biomarker in HCC and may facilitate the development of targeted precision oncology. Moreover, these results were reconfirmed with our analyses of HCC sample groups. Again, we found no significant correlation between CCDC6 expression and iCCA group with the patients data from our hospital (**Figure 3**). Francesco and colleagues presented evidence for the downregulation of CCDC6 protein enhancing tumor aggressiveness and reducing sensitivity to DNA damaging agents, such as cisplatinum, in patients with non-small cell lung cancer (NSCLC). But interestingly, CCDC6 could sensitize the cells to olaparib, a small molecule inhibitor of the repair enzymes PARP1/2 26. Therefore, these authors proposed CCDC6 as a predictive biomarker for PARP1 targeting therapy, and they showed that a low CCDC6 protein expression (in 51 out of 138 patients) was correlated with lymph node positivity, DFS and OS in the patients with NSCLC, a finding differing from ours in the HCC group. A study on the association between CCDC6 and gastric cancer found that CCDC6 was highly expressed in gastric cancer, compared with the expression in normal gastric tissues 27. Moreover, a significantly positive correlation between CCDC6 gene expression levels and the microsatellite instability (MSI) score was also reported for gastric cancer. However, their following investigation on TCGA database revealed a lack of significant correlations between CCDC6 and clinical variables (including the age, sex, pathological stage, tumor size, T classification, N classification, distant metastasis, or pathological grade) of patients with in gastric cancer. Still, their online Kaplan-Meier analysis showed that the patients with gastric cancer and high CCDC6 expression had shorter OSs after chemotherapy 27. These findings are similar to ours in HCC. Current studies have confirmed the CCDC6 gene as a tumor suppressor gene in non-small cell lung cancer 26 and thyroid cancer^28^, but our research data suggests the possibility that CCDC6 may be a proto-oncogene, especially in HCC. The analysis data from the HPA database (https://www.proteinatlas.org/) also has revealed that CCDC6 can be a negative prognostic marker for liver and pancreatic cancers, but that it can also be a positive prognostic marker for head and neck cancer. The role of CCDC6 as a tumor suppressor gene in different types of cancers remains unclear. Our data from immunohistochemical and online databases indicate that the high expression of CCDC6 is associated with a poor prognosis in patients with HCC.

### CCDC6 is associated with histone acetylation in HCC

Known to be a cancer driver gene, CCDC6 (coiled-coil domain containing protein 6) is expressed as a 55 KDa nuclear and cytosolic protein involved in apoptosis as well as the DNA damage response. When DNA damage occurs in normal cells, CCDC6 controls the cellular checkpoints of DNA damage recovering, so as to maintain the cell cycle and genomic stability or otherwise promote apoptosis 29. Loss of CCDC6 has been shown to result in increased cell death with clear shortening of the S phase transition of the cell cycle 30. CCDC6 must be kept in the nucleus to work efficiently. If it comes out of the nucleus somehow, then it cannot work properly, which leads to cancer 31. On the basis of the tight correlation between CCDC6 expression and hepatobiliary tumors, especially HCC, we conducted the following investigations on different online databases. Based on GeneMania and String database, we found correlations between the CCDC6 gene and its protein products and PPP4C, PPP4R1, PPP2R1A, and CUX1, all of which are related to histone acetylation (**Figure 4**). Moreover, GO, KEGG, GSEA and GSVA analyses revealed similar terms of histone acetylation and cell cycle phase transition (**Figures 5-6**). With the elimination of the electron force between histones and DNA, the stability change of nucleosomes means acetylation can directly help DNA transcription, replication and repair mechanisms 32. Protein serine/threonine phosphatase 4 (PPP4C) is an essential polypeptide involved in critical cellular processes such as microtubule growth and organization, DNA damage checkpoint recovery, apoptosis, and tumor necrosis factor alpha signaling 33. The absence of CCDC6 function may affect the genome stability, leading to carcinogenesis. Recent research has demonstrated CCDC6's interaction with PPP4C negatively modulating the phosphatase enzymatic activity toward the dephosphorylation on S139 of the histone H2AX (γH2AX), the specific marker and efficient coordinator of the DNA repairing process. In primary tumors the loss of CCDC6 function could influence genome stability thereby contributing to carcinogenesis 10. Aberrant epigenetic silencing of tumor suppressor genes by promoter DNA hyper-methylation and histone deacetylation has an important role in carcinogenesis. The potential reversibility of these epigenetic abnormalities makes targeting them with drugs that modify chromatin an attractive therapeutic approach 34. The investigation of inhibitors of DNA methyltransferase (DNMT) and histone deacetylase (HDAC) is a hot spot in epigenetics.

### Carcinogenicity of CCDC6 fusion mutation

The number of CCDC6 molecular alterations identified has grown in human cancers. Multiple partner genes in fusions contribute functionally to the activity of known oncogenes like RET, MYC, MLL, and others. Especially in papillary thyroid carcinoma with the fusion of CCDC6 and RET, CCDC6 has been reported to interact with CREB1 (cAMP response element binding protein 1, a protein involved in the regulation of thyroid cell proliferation) and repress its transcriptional activity by recruiting histone deacetylase 1 and protein phosphatase 1 proteins at the CRE site of the CREB1 target genes 28. But the fusion of CCDC6 and RET in thyroid cancer cells abrogates the ability to combine with CREB1 so as to activate CREB1. Considering that higher CCDC6 expression levels are associated with a poor prognosis of patients with HCC in our study, we speculate that when CCDC6 is fused with other proto-oncogenes, the expression of a residual wild-type allele and the expression of fused proto-oncogenes may both contribute to tumor development 29. We hypothesize that the folding of the fusion protein may improve through protein dimerization and oncogene activation via the coiled-coil region, but the hypothetical mechanism still needs experimental clarification. Zofia and colleagues revealed that CCDC6-RET fusions can mediate acquired resistance to EGFR tyrosine kinase inhibitors and that combined EGFR and RET inhibition may be a well-tolerated and effective treatment strategy for patients with NSCLC and acquired CCDC6-RET fusions 35.

### Correlation between CCDC6 and immune infiltrating cells and its potential as a predictive biomarker in targeted therapy

The tumor microenvironment (TME) of HCC is a complex and spatially structured mixture of hepatic non-parenchymal resident cells, tumor cells, immune cells and tumor-associated fibroblasts 36. Immune checkpoint blockade takes advantage of the immune cell infiltration in the tumor to reinvigorate an efficacious antitumoral immune response 37. Owing to the principle, many immunotherapy drugs have had roles in the treatment of HCC, such as nivolumab and pembrolizumab. The composition of the TME influences the response to immune checkpoint blockade. Here, we report that high CCDC6 expression in HCC is correlated with increased infiltration by B cells, CD4+ T cells, CD8+ T cells, neutrophils, macrophages, and dendritic cells (**Figure 7**). Moreover, we observed a significant association between CCDC6 and various immune cell marker sets in HCC (**Tables 1 and 2**). CCDC6 expression was also positively correlated with PD-L1 and CTLA-4 expressions (**Figure 7**). Interestingly, though we did not find a close association between CCDC6 expression and the types of immune cells infiltrating CCA from TIMER, we did find a weak association between CCDC6 and PD-1/PDCD1/CTLA4 in CCA from GEPIA. However, Fabris and colleagues have revealed a role for the TME containing fibrogenic cells, lymphatics and a variety of immune cells in CCA progression 38. Our data revealed the correlation between CCDC6 expression and immune cells in HCC. It could be seen that the expression of CCDC6 could be detected in various cell types in the liver, and the first four of them were Kupfer cells, cholangiocytes, endothelial cells and hepatocytes (HPA database). And Kupfer cell was an important cellular component of the liver immune system. By exploring the expression of CCDC6 in various types of immune cells in HPA database, we could also find that CCDC6 was mainly expressed in Myeloid DC, B-cell, monocyte and T-cell. These results were consistent with our research. A recent study also detected a CCDC6-RET fusion in all 4 male patients with positive expression of PD-L1 39. Japanese researchers also reported the beneficial effects of pembrolizumab in a patient with PD-L1+ lung adenocarcinoma, while the CCDC6-RET fusion gene and co-occurring NF1/TP53 mutations were also detected 40. In view of their small group of cases, these findings only suggest that CCDC6 may be a novel immune-related therapeutic target in HCC. However, deeper exploration is necessary to Figure out the precise role of CCDC6 in the tumor-immune microenvironment. As mentioned above, CCDC6 with mutations confers resistance to chemotherapeutic agents and sensitivity to small molecule inhibitors of the repair enzymes PARP1/2. In the meanwhile, PARP inhibitors can promote the immune priming of the tumor by increasing the neoantigen exposure and the upregulation of programmed death ligand 1 (PD-L1) expression 41. Considering this circumstance, combing molecularly-targeted therapy (PARP inhibitors), epigenetic drugs (HDAC inhibitors) and immunotherapy may amplify the curative effects of each single drug, while reducing their doses and toxicity 42, 43. In addition, CCDC6 may be used as a molecular predictor for the prognosis of this kind of comprehensive therapy in patients with CCDC6 mutations. The benefits of this comprehensive therapy (also known as stereotactic therapy) have been apparent for a patient with HCC in our clinical practice (**Supplementary Figure 7**) 44,45. Clinical trials of single or combination therapy with PARP inhibitors, immune checkpoint inhibitors and epigenetic drugs for ovarian, breast, pancreatic, and lung cancers are underway. Our research results suggest that CCDC6 expression was increased in hepatobiliary carcinoma. The over expressions of CCDC6 were found to be significantly associated with clinical parameters (especially in clinical cancer stages and pathological tumor grades) and poor prognosis of HCC patients. Its upregulation is associated with histone acetylation and immune infiltration in hepatobiliary carcinoma.

## Limitations

We performed a comprehensive and systematic analysis on CCDC6 and used different databases, R 3.2.2, and IHC for cross-verification, but some limitations persist in our study. First, the microarray and sequencing data from different databases exhibited differences, which might cause systematic bias. Second, *in vivo*/*in vitro* experiments are needed to confirm our results on the potential functions of CCDC6. Third, even though we concluded that CCDC6 expression was strongly related to histone acetylation, immune cell infiltration and prognosis of HCC, we lack direct evidence on CCDC6 influencing prognosis by playing a role in histone acetylation and/or immune infiltration. Online databases currently lack a detailed classification of CCA into iCCA and non-iCCA; therefore, we decided to use CCA as a whole in our research. Thus, we were not able to explore the mechanisms by which CCDC6 participates in histone acetylation and the immune system response, and the pathways need further study.

In conclusion, our results revealed that over expressions of CCDC6 is significantly associated with clinical cancer stages and pathological tumor grades in patients with HCC.

## Conclusion

In conclusion, we found higher CCDC6 expressions to be significantly associated with OS in both online databases and in our cohort. Multivariate analysis of our own patient data also showed that higher expressions of CCDC6 were independent prognostic factors for shorter OSs in the patients with HCC. Moreover, CCDC6 expression seems to be tightly associated with histone acetylation and immune infiltrations. These results indicate that CCDC6 may be a prognostic biomarker for HCC survival and to predict comprehensive therapy outcomes in patients with CCDC6 mutations.

## Supplementary Material

Supplementary figures.

## Figures and Tables

**Table 1:** Correlation between CCDC6 expression and clinicopathological variables in 94 HCC cases

Clinicopathological variables	Number of each group (%)	CCDC6 expression		P value
		Low (33)	High (61)	
**Age (years)**				0.933
<50	29 (30.9)	10	19	
≥50	65 (69.1)	23	42	
**Gender**				0.732
Male	84 (89.4)	29	55	
Female	10 (10.6)	4	6	
**Tumor Volume**				0.829
<125CM3	47 (50)	17	30	
≥125CM3	47 (50)	16	31	
**Differentiation Grade**				**0.005**
high	24 (25.6)	15	9	
moderate	35 (37.2)	8	27	
low/none	35 (37.2)	10	25	
**Cirrhosis**				0.077
No	54 (57.4)	23	31	
Yes	40 (42.6)	10	30	
**Invasion of local organs or lymph nodes**				0.054
No	79 (84.0)	31	48	
Yes	15 (16.0)	2	13	
**AJCC clinical stage**				0.038
I-II	49 (52.1)	22	27	
III-IV	45 (47.9)	11	34	
**Death**				**0.001**
Yes	34 (36.2)	19	15	
No	60 (63.8)	14	46	

**Table 2:** Correlation between CCDC6 expression and clinicopathological variables in 99 iCCA cases

Clinicopathological variables	Number of each group (%)	CCDC6 expression		P value
		Low (47)	High (52)	
**Age (years)**				0.970
<50	17 (17.2)	8	9	
≥50	82 (82.8)	39	43	
**Gender**				0.704
Male	63 (63.7)	29	34	
Female	36 (36.3)	18	18	
**Tumor Diameter**				0.147
<5CM	25 (25.2)	15	10	
≥5CM	74 (74.8)	32	42	
**Differentiation Grade**				0.850
High/moderate	58 (58.6)	28	30	
Low/none	41 (41.4)	19	22	
**Invasion of local organs or lymph nodes**				0.324
No	74 (74.8)	33	41	
Yes	25 (25.2)	14	11	
**AJCC clinical stage**				0.210
I	58 (58.6)	27	31	
II	18 (18.2)	6	12	
III-IV	23 (23.2)	14	9	
**Tumor location**				0.473
Left	37 (37.4)	19	18	
Middle	33 (33.3)	17	16	
Right	29 (29.3)	11	18	
Death	33 (100)			0.876
Yes	7 (21.2)	4	3	
No	26 (78.8)	14	12	

**Table 3:** Univariable and multivariable analysis of overall survival in HCC patients

Variables	Univariate analysis			Multivariate analysis		
	HR	95%CI	*p* value	HR	95%CI	*p* value
CCDC6 expression (low vs. high)	2.593	1.420-4.736	0.002	1.967	1.064-3.634	0.031
Age (<45 vs. ≥45)	0.730	0.4263-1.251	0.252			
Gender (male vs. female)	0.678	0.271-1.696	0.406			
Differentiation Grade	1.364	0.697-2.668	0.365			
AJCC Clinical stage (I + II vs. III + IV)	4.473	2.551-7.843	<0.001	3.908	2.210-6.912	<0.001
Tumor volume (<125 vs. ≥125)	1.892	1.126-3.176	0.016			
Invasion of local organs or lymph nodes (yes vs. no)	3.917	2.113-7.260	<0.001			
cirrhosis (yes vs. no)	1.073	0.643-1.792	0.787			

**Table 4:** Univariable and multivariable analysis of overall survival in iCCA patients

Variables	Univariate analysis			Multivariate analysis		
	HR	95%CI	*p* value	HR	95%CI	*p* value
CCDC6 expression (low vs. high)	0.549	0.251-1.200	0.133	0.141	0.050-0.399	<0.001
Age (<50 vs. ≥50)	0.977	0.127-7.526	0.982			
Gender (male vs. female)	0.978	0.437-2.188	0.956			
Differentiation Grade (high/moderate vs. low/none)	0.802	0.344-1.867	0.608			
AJCC Clinical stage (I + II vs. III + IV)	2.375	1.051-5.369	0.038	2.818	1.207-6.581	0.017
Tumor diameter (<5 vs. ≥5)	3.239	1.210-8.671	0.019	11.333	3.122-41.146	<0.001
Invasion of local organs or lymph nodes (yes vs. no)	3.917	2.113-7.260	0.238			
Tumor location (right, middle and left )	0.419	0.099-1.779	0.238			

**Table 5:** Correlation analysis between CCDC6 expression and related markers of immune cells using TIMER database

Description	Gene markers	LIHC				CHOL			
		None		Purity		None		Purity	
		Cor	P	Cor	P	Cor	P	Cor	P
CD8+ T cell	CD8A	0.243	***	0.258	***	0.023	ns	0.077	ns
	CD8B	0.145	**	0.17	**	-0.129	ns	-0.1	ns
	CD45 (PTPRC)	0.476	***	0.493	***	0.183	ns	0.314	ns
T cell (general)	CD3D	0.168	**	0.185	***	0.19	ns	0.285	ns
	CD3E	0.229	***	0.242	***	0.124	ns	0.229	ns
	CD2	0.207	***	0.226	***	0.071	ns	0.155	ns
B cell	CD19	0.207	***	0.207	***	0.155	ns	0.241	ns
	CD79A	0.189	***	0.197	***	0.18	ns	0.262	ns
	CD27	0.206	***	0.231	***	0.07	ns	0.14	ns
	CD20 (MS4A1)	0.174	***	0.17	**	0.107	ns	0.178	ns
Monocyte	CD14	-0.325	***	-0.318	***	-0.172	ns	-0.139	ns
	CD86	0.372	***	0.392	***	0.031	ns	0.101	ns
	CD115 (CSF1R)	0.316	***	0.337	***	-0.027	ns	0.015	ns
TAM	CCL2	0.252	***	0.258	***	0.127	ns	0.156	ns
	CD68	0.322	***	0.327	***	-0.106	ns	-0.08	ns
	IL10	0.317	***	0.321	***	0.065	ns	0.182	ns
M1 Macrophage	INOS (NOS2)	0.194	***	0.191	***	0.156	ns	0.158	ns
	CD80	0.392	***	0.412	***	-0.089	ns	-0.045	ns
	IRF5	0.444	***	0.446	***	0.025	ns	0.051	ns
	IL6	0.153	**	0.169	**	0.234	ns	0.358	*
	PTGS2	0.362	***	0.384	***	0.222	ns	0.281	ns
	CD64 (FCGR1A)	0.31	***	0.334	***	0.175	ns	0.232	ns
M2 Macrophage	CD163	0.255	***	0.269	***	0.34	*	0.451	**
	CD206 (MRC1)	0.104	*	0.123	*	0.07	ns	0.122	ns
	VSIG4	0.248	***	0.271	***	0.141	ns	0.209	ns
	MS4A4A	0.255	***	0.279	***	0.215	ns	0.356	*
Neutrophils	CD66b (CEACAM8)	0.109	*	0.12	*	0.197	ns	0.2	ns
	CD11b (ITGAM)	0.36	***	0.391	***	0.008	ns	0.035	ns
	CCR7	0.247	***	0.252	***	0.147	ns	0.254	ns
	CD15 (FUT4)	0.552	***	0.561	***	0.458	**	0.476	**
Natural killer cell	KIR2DL1	0.008	ns	0.001	ns	-0.047	ns	-0.033	ns
	KIR2DL3	0.172	***	0.191	***	0.037	ns	0.049	ns
	KIR2DL4	0.116	*	0.126	*	-0.163	ns	-0.144	ns
	KIR3DL1	0.086	ns	0.106	*	-0.157	ns	-0.145	ns
	KIR3DL2	0.094	ns	0.117	*	0.045	ns	0.048	ns
	KIR3DL3	-0.02	ns	-0.043	ns	0.026	ns	0.041	ns
	CD56 (NCAM1)	0.341	***	0.37	***	0.18	ns	0.194	ns
	CD335 (NCR1)	0.18	***	0.193	***	0.304	ns	0.402	*
Dendritic cell	BDCA-1 (CD1C)	0.297	***	0.298	***	-0.028	ns	0.02	ns
	HLA-DPB1	0.268	***	0.283	***	-0.111	ns	-0.074	ns
	HLA-DQB1	0.166	**	0.187	***	0.025	ns	0.057	ns
	HLA-DRA	0.306	***	0.321	***	-0.067	ns	-0.02	ns
	HLA-DPA1	0.3	***	0.321	***	-0.081	ns	-0.037	ns
	BDCA-3 (CD141) (THBD)	0.275	***	0.269	***	0.298	ns	0.374	*
	BDCA-4 (NRP1)	0.563	***	0.568	***	0.22	ns	0.27	ns
	CD123 (IL3RA)	0.065	ns	0.07	ns	0.065	ns	0.127	ns
	CD11c (ITGAX)	0.405	***	0.419	***	-0.005	ns	0.054	ns
Th1	T-bet (TBX21)	0.144	**	0.158	**	0.023	ns	0.106	ns
	STAT4	0.273	***	0.29	***	0.13	ns	0.17	ns
	STAT1	0.481	***	0.491	***	0.466	**	0.493	**
	TNF	0.315	***	0.325	***	0.051	ns	0.07	ns
	IFNG	0.188	***	0.211	***	-0.164	ns	-0.132	ns
Th2	GATA3	0.324	***	0.345	***	-0.103	ns	-0.056	ns
	STAT6	0.384	***	0.376	***	0.432	**	0.431	**
	IL13	0.042	ns	0.036	ns	-0.024	ns	0.002	ns
	STAT5A	0.425	***	0.444	***	0.049	ns	0.07	ns
Tfh	BCL6	0.325	***	0.322	***	0.162	ns	0.172	ns
	IL21	0.136	**	0.147	**	0.039	ns	0.07	ns
Th17	STAT3	0.471	***	0.483	***	0.223	ns	0.226	ns
	IL17A	0.125	*	0.114	*	0.08	ns	0.108	ns
Treg	FOXP3	0.228	***	0.238	***	-0.027	ns	0.031	ns
	CD25 (IL2RA, ISG20)	-0.012	ns	-0.003	ns	-0.019	ns	-0.019	ns
	CCR8	0.479	***	0.498	***	0.107	ns	0.163	ns
	STAT5B	0.554	***	0.557	***	0.314	ns	0.329	ns
	TGFB1	0.469	***	0.484	***	0.15	ns	0.189	ns
Exhausted T cell	PD-1 (PDCD1)	0.28	***	0.279	***	0.222	ns	0.261	ns
	CTLA4	0.236	***	0.253	***	0.118	ns	0.161	ns
	LAG3	0.113	*	0.117	*	-0.049	ns	-0.011	ns
	TIM-3 (HAVCR2)	0.373	***	0.4	***	-0.018	ns	0.034	ns
	CXCL13	0.203	***	0.22	***	0.18	ns	0.248	ns
	LAYN	0.431	***	0.454	***	0.089	ns	0.114	ns
Resting Treg	FOXP3	0.228	***	0.238	***	-0.027	ns	0.031	ns
	IL2RA	0.327	***	0.336	***	0.252	ns	0.346	*
Effector Treg T-cell	FOXP3	0.228	***	0.238	***	-0.027	ns	0.031	ns
	CCR8	0.479	***	0.498	***	0.107	ns	0.163	ns
	TNFRSF9	0.424	***	0.46	***	0.015	ns	0.086	ns
Effector T-cell	CX3CR1	0.474	***	0.485	***	0.085	ns	0.122	ns
	FGFBP2	-0.035	ns	-0.024	ns	-0.202	ns	-0.187	ns
	FCGR3A	0.312	***	0.337	***	0.14	ns	0.182	ns
Naïve T-cell	CCR7	0.247	***	0.252	***	0.147	ns	0.254	*
	SELL	0.277	***	0.301	***	0.111	ns	0.206	ns
Effector memory T-cell	DUSP4	0.418	***	0.445	***	0.129	ns	0.127	ns
	GZMK	0.12	*	0.134	*	0.097	ns	0.199	ns
	GZMA	0.082	ns	0.105	ns	0.06	ns	0.124	ns
Resident memory T-cell	CD69	0.306	***	0.32	***	0.221	ns	0.323	ns
	CXCR6	0.225	***	0.245	***	0.077	ns	0.164	ns
	MYADM	0.612	***	0.61	***	0.464	**	0.473	**
General memory T-cell	CCR7	0.247	***	0.252	***	0.147	ns	0.254	*
	SELL	0.277	***	0.301	***	0.111	ns	0.206	ns
	IL7R	0.373	***	0.385	***	0.285	ns	0.389	*

## Abbreviations

CCDC6: Coiled-coil domain containing 6
HCC: Hepatocellular carcinoma
LIHC: Liver hepatocellular carcinoma
CCA/CHOL: Cholangiocarcinoma
iCCA: Intrahepatic Cholangiocarcinoma
DSS: disease-specific survival
DFI: disease-free interval
PFI: progression-free interval
OS: overall survival
