# Supplementary material for: High expression of CCDC6 in relation to unfavorable outcome and immune cells infiltration in hepatobiliary carcinoma
Source: J Cancer. 2022 Sep 21;13(12):3378–95. doi: 10.7150/jca.76050 (PMC9516016; doi:10.7150/jca.76050)
Supplement: Supplementary file 1 — Supplementary figures. [file jcav13p3378s1.pdf]

Figure S1. CCDC6 expression in HCC based on TP53 mutation status and the DFI curves in HCC/CCA based on CCDC6 expression level (A)The correlation between CCDC6 and TP53 mutation of HCC according to UALCAN database. (B-C) the DFI curves in HCC/CCA based on CCDC6 expression level according to TCGA database.

Figure S2. Representative IHC-stained slides of HCC/iCCA from the Human Protein Atlas (HPA). (A-B) Representative IHC-stained slides of HCC. (C). Representative IHC-stained slides of HCC. (D) Representative IHC-stained slides of normal liver tissue.

Figure S3. The top 50 negative genes in HCC and CCA

Figure S4. GO and KEGG enrichment analysis for CCDC6. (A-B) Top 20 enrichment terms in CC and MF categories in HCC. (C-D) Top 20 enrichment terms in CC and MF categories in CCA.

Figure S5. Correlation of CCDC6 expression with immune infiltration cells in HCC and CCA (A) CCDC6 is positively correlated with the infiltration of Dendritic.Cells, Dendritic.Cells.Resting, Macrophages.M0, Mast.Cells, Mast.Cells.Resting, Monocytes, NK.Cells.Resting , T.Cells.CD4.Memory.Resting and T.Cells.CD4.Naive in HCC using the TCGA database. (B) CCDC6 is positively correlated with the infiltration of NK.Cells.Resting, T.Cells.CD4.Memory.Resting and T.Cells.CD4.Naive in CCA using the TCGA database.

Figure S6. GSEA analysis (Reactomeresult and KEGG) of HCC(A,B) and CCA(C,D)

Figure S7. One typical HCC patient confirmed CCDC6 positive got complete response after receiving targeted therapy, immunotherapy, TACE and surgery. (A). Enhanced CT-scan revealed a major tumor mass before cure (white arrow). (B). Enhanced CT-scan revealed another two small masses (metastasis， white arrow) before cure.(C). IHC of the tumor tissue according liver biopsy. (D). Enhanced CT-scan revealed tumor regression after comprehensive therapy. (E). Enhanced CT-scan revealed no reoccurrence of tumor after surgery (7 months). (i, routine scan, ii, arterial phase, iii, venous phase, iv, delayed phase)

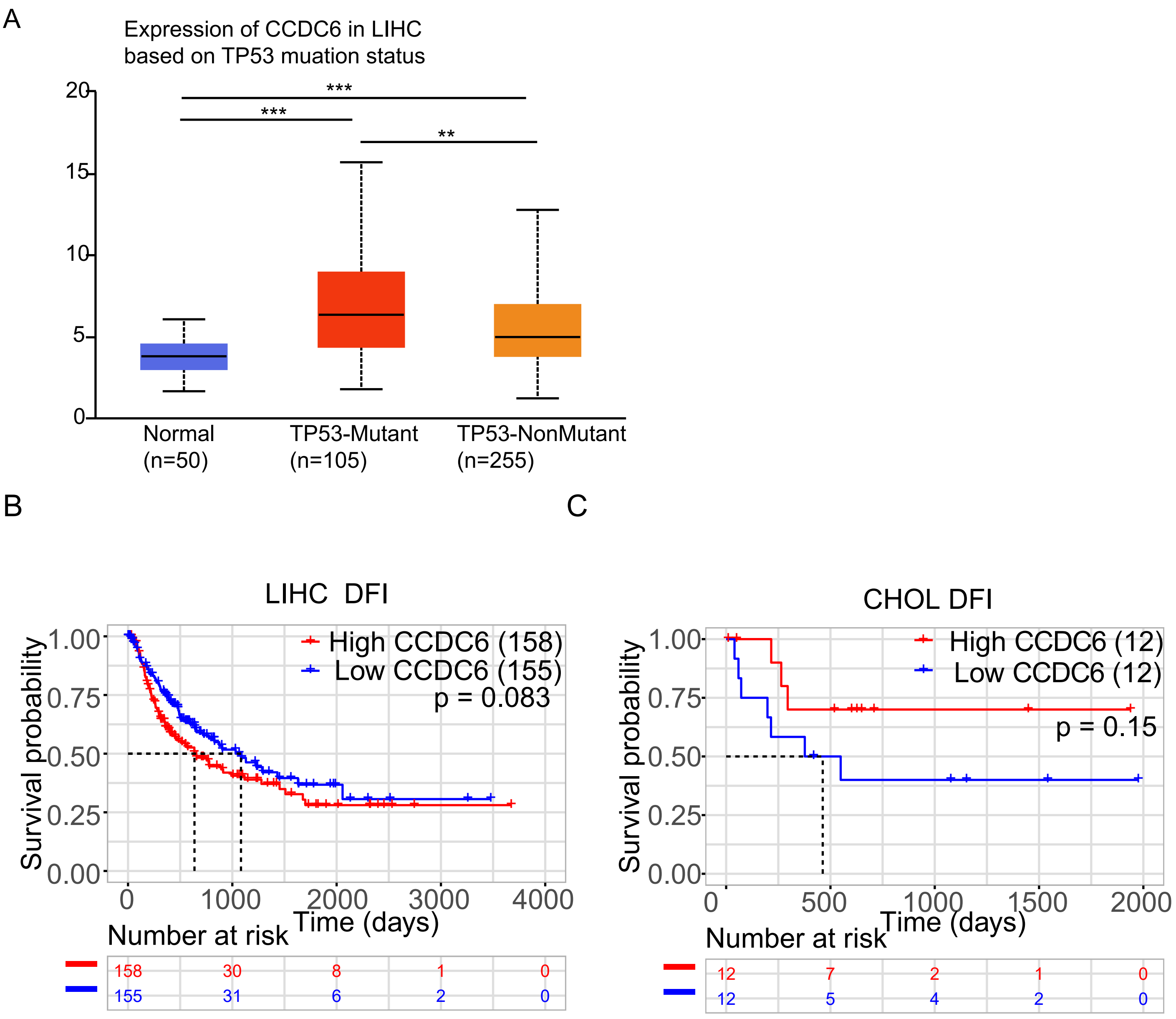
A
Expression of CCDC6 in LIHC
based on TP53 muation status
***
***
**
20
15
10
5
0
Normal
(n=50)
TP53-Mutant
(n=105)
TP53-NonMutant
(n=255)
B
LIHC DFI
High CCDC6 (158)
Low CCDC6 (155)
p = 0.083
Survival probability
1.00
0.75
0.50
0.25
0.00
0
1000
2000
3000
4000
Time (days)
Number at risk
158 30 8 1 0
155 31 6 2 0
C
CHOL DFI
High CCDC6 (12)
Low CCDC6 (12)
p = 0.15
Survival probability
1.00
0.75
0.50
0.25
0.00
0
500
1000
1500
2000
Time (days)
Number at risk
12 7 2 1 0
12 5 4 2 0

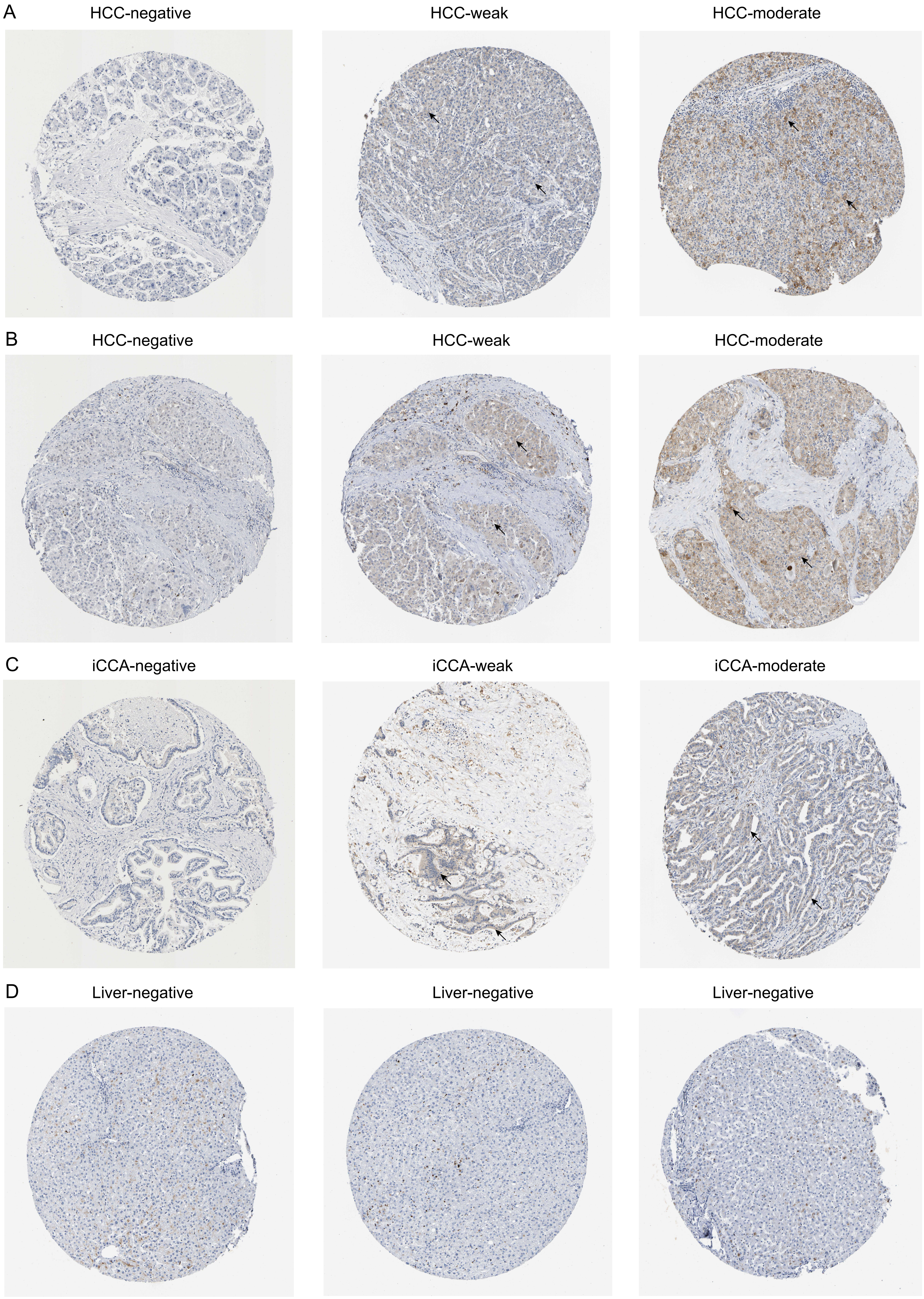

A
HCC-negative
HCC-weak
HCC-moderate
B
HCC-negative
HCC-weak
HCC-moderate
C
iCCA-negative
iCCA-weak
iCCA-moderate
D
Liver-negative
Liver-negative
Liver-negative

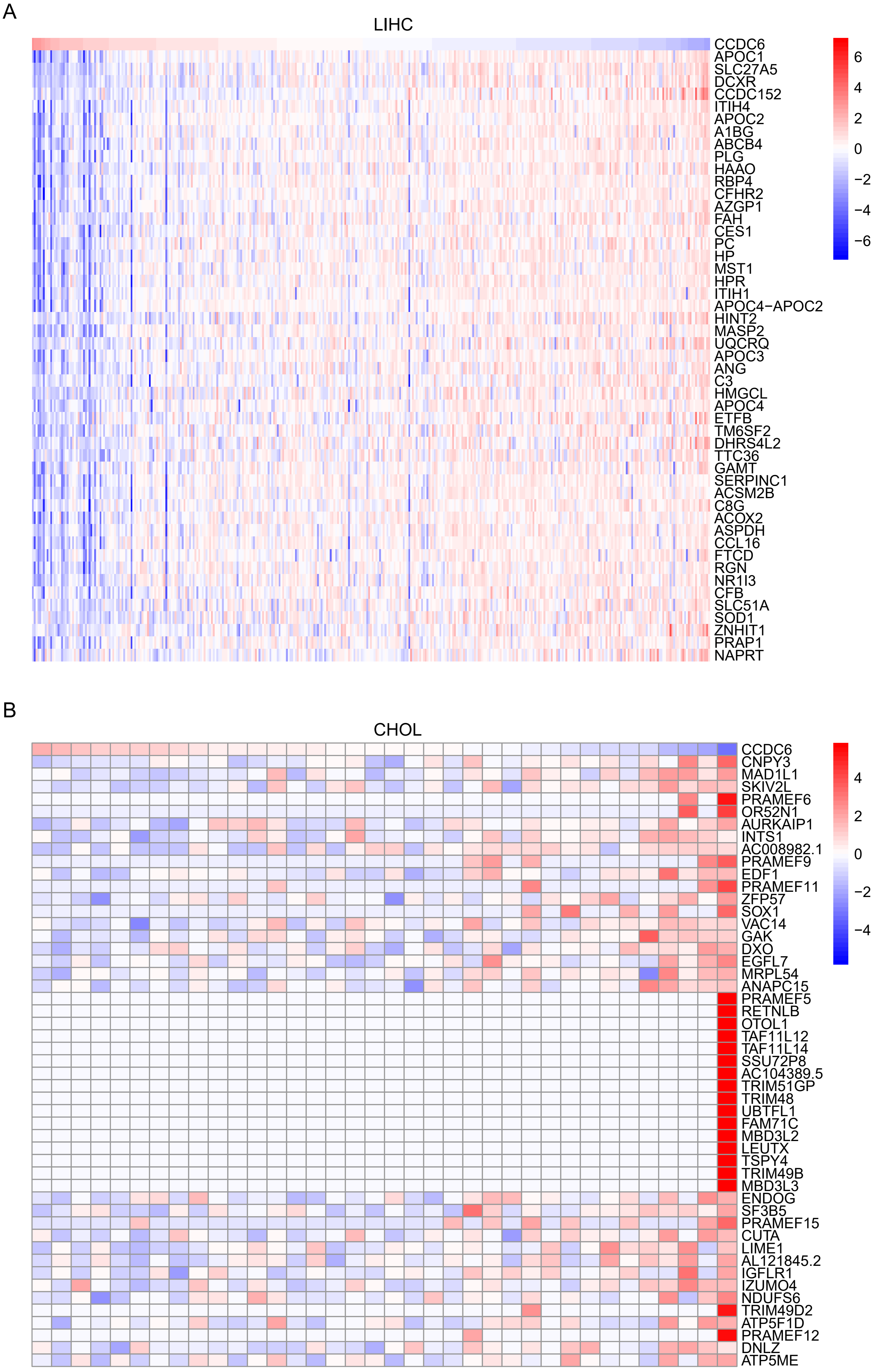
A
LIHC
CCDC6
APOC1
SLC27A5
DCXR
CCDC152
ITIH4
APOC2
A1BG
ABCB4
PLG
HAAO
RBP4
CFHR2
AZGP1
FAH
CES1
PC
HP
MST1
HPR
ITIH1
APOC4−APOC2
HINT2
MASP2
UQCRQ
APOC3
ANG
C3
HMGCL
APOC4
ETFB
TM6SF2
DHRS4L2
TTC36
GAMT
SERPINC1
ACSM2B
C8G
ACOX2
ASPDH
CCL16
FTCD
RGN
NR1I3
CFB
SLC51A
SOD1
ZNHIT1
PRAP1
NAPRT
6
4
2
0
−2
−4
−6
B
CHOL
CCDC6
CNPY3
MAD1L1
SKIV2L
PRAMEF6
OR52N1
AURKAIP1
INTS1
AC008982.1
PRAMEF9
EDF1
PRAMEF11
ZFP57
SOX1
VAC14
GAK
DXO
EGFL7
MRPL54
ANAPC15
PRAMEF5
RETNLB
OTOL1
TAF11L12
TAF11L14
SSU72P8
AC104389.5
TRIM51GP
TRIM48
UBTFL1
FAM71C
MBD3L2
LEUTX
TSPY4
TRIM49B
MBD3L3
ENDOG
SF3B5
PRAMEF15
CUTA
LIME1
AL121845.2
IGFLR1
IZUMO4
NDUFS6
TRIM49D2
ATP5F1D
PRAMEF12
DNLZ
ATP5ME
4
2
0
−2
−4

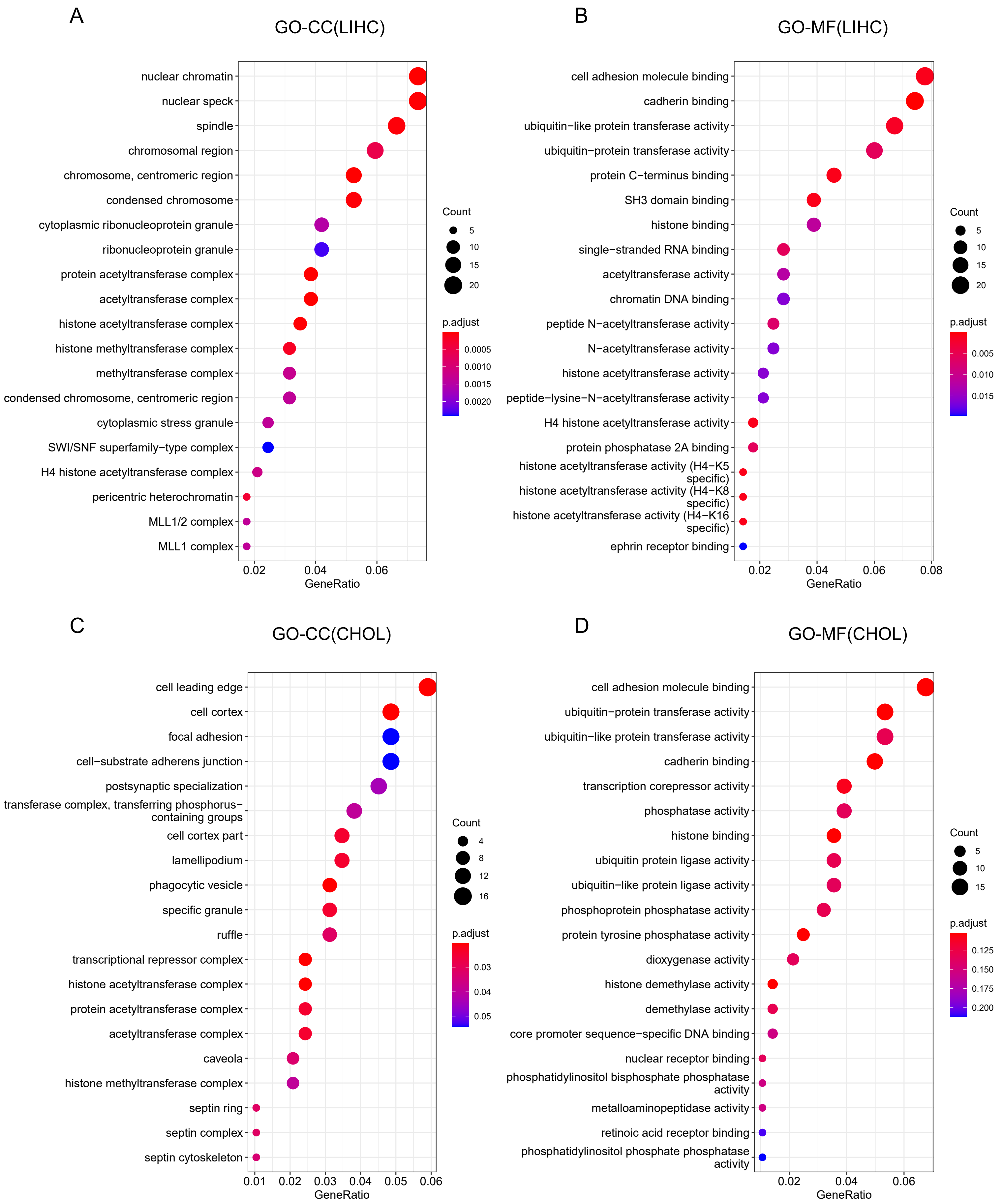

A
GO-CC(LIHC)
nuclear chromatin
nuclear speck
spindle
chromosomal region
chromosome, centromeric region
condensed chromosome
cytoplasmic ribonucleoprotein granule
ribonucleoprotein granule
protein acetyltransferase complex
acetyltransferase complex
histone acetyltransferase complex
histone methyltransferase complex
methyltransferase complex
condensed chromosome, centromeric region
cytoplasmic stress granule
SWI/SNF superfamily−type complex
H4 histone acetyltransferase complex
pericentric heterochromatin
MLL1/2 complex
MLL1 complex
0.02
0.04
0.06
GeneRatio
Count
5
10
15
20
p.adjust
0.0005
0.0010
0.0015
0.0020
B
GO-MF(LIHC)
cell adhesion molecule binding
cadherin binding
ubiquitin−like protein transferase activity
ubiquitin−protein transferase activity
protein C−terminus binding
SH3 domain binding
histone binding
single−stranded RNA binding
acetyltransferase activity
chromatin DNA binding
peptide N−acetyltransferase activity
N−acetyltransferase activity
histone acetyltransferase activity
peptide−lysine−N−acetyltransferase activity
H4 histone acetyltransferase activity
protein phosphatase 2A binding
histone acetyltransferase activity (H4−K5 specific)
histone acetyltransferase activity (H4−K8 specific)
histone acetyltransferase activity (H4−K16 specific)
ephrin receptor binding
0.02
0.04
0.06
0.08
GeneRatio
Count
5
10
15
20
p.adjust
0.005
0.010
0.015
C
GO-CC(CHOL)
cell leading edge
cell cortex
focal adhesion
cell−substrate adherens junction
postsynaptic specialization
transferase complex, transferring phosphorus−containing groups
cell cortex part
lamellipodium
phagocytic vesicle
specific granule
ruffle
transcriptional repressor complex
histone acetyltransferase complex
protein acetyltransferase complex
acetyltransferase complex
caveola
histone methyltransferase complex
septin ring
septin complex
septin cytoskeleton
0.01
0.02
0.03
0.04
0.05
0.06
GeneRatio
Count
4
8
12
16
p.adjust
0.03
0.04
0.05
D
GO-MF(CHOL)
cell adhesion molecule binding
ubiquitin−protein transferase activity
ubiquitin−like protein transferase activity
cadherin binding
transcription corepressor activity
phosphatase activity
histone binding
ubiquitin protein ligase activity
ubiquitin−like protein ligase activity
phosphoprotein phosphatase activity
protein tyrosine phosphatase activity
dioxygenase activity
histone demethylase activity
demethylase activity
core promoter sequence−specific DNA binding
nuclear receptor binding
phosphatidylinositol bisphosphate phosphatase activity
metalloaminopeptidase activity
retinoic acid receptor binding
phosphatidylinositol phosphate phosphatase activity
0.02
0.04
0.06
GeneRatio
Count
5
10
15
p.adjust
0.125
0.150
0.175
0.200

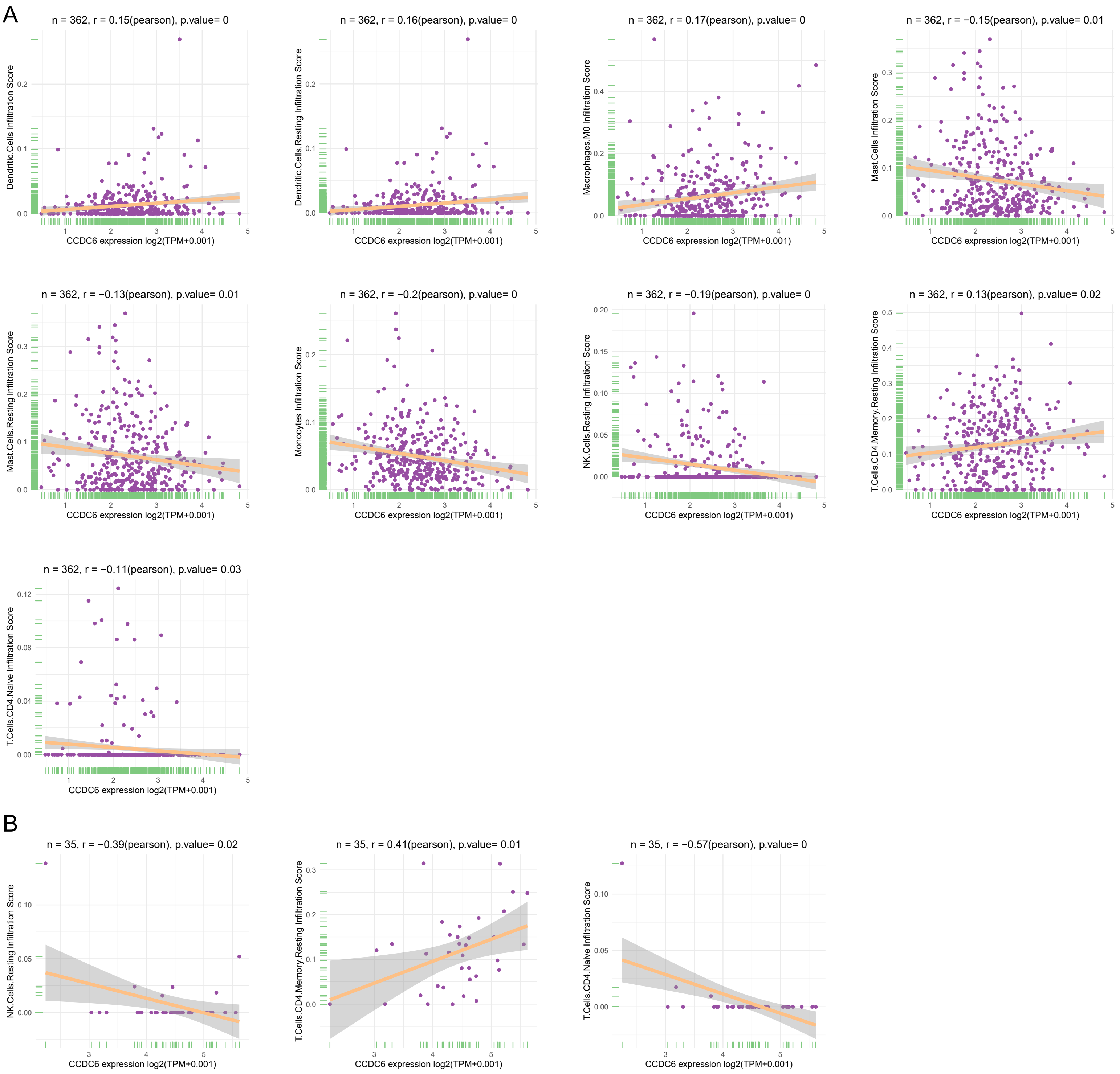
A
n = 362, r = 0.15(pearson), p.value= 0
Dendritic.Cells Infiltration Score
CCDC6 expression log2(TPM+0.001)
n = 362, r = 0.16(pearson), p.value= 0
Dendritic.Cells.Resting Infiltration Score
CCDC6 expression log2(TPM+0.001)
n = 362, r = 0.17(pearson), p.value= 0
Macrophages.M0 Infiltration Score
CCDC6 expression log2(TPM+0.001)
n = 362, r = −0.15(pearson), p.value= 0.01
Mast.Cells Infiltration Score
CCDC6 expression log2(TPM+0.001)
n = 362, r = −0.13(pearson), p.value= 0.01
Mast.Cells.Resting Infiltration Score
CCDC6 expression log2(TPM+0.001)
n = 362, r = −0.2(pearson), p.value= 0
Monocytes Infiltration Score
CCDC6 expression log2(TPM+0.001)
n = 362, r = −0.19(pearson), p.value= 0
NK.Cells.Resting Infiltration Score
CCDC6 expression log2(TPM+0.001)
n = 362, r = 0.13(pearson), p.value= 0.02
T.Cells.CD4.Memory.Resting Infiltration Score
CCDC6 expression log2(TPM+0.001)
n = 362, r = −0.11(pearson), p.value= 0.03
T.Cells.CD4.Naive Infiltration Score
CCDC6 expression log2(TPM+0.001)
B
n = 35, r = −0.39(pearson), p.value= 0.02
NK.Cells.Resting Infiltration Score
CCDC6 expression log2(TPM+0.001)
n = 35, r = 0.41(pearson), p.value= 0.01
T.Cells.CD4.Memory.Resting Infiltration Score
CCDC6 expression log2(TPM+0.001)
n = 35, r = −0.57(pearson), p.value= 0
T.Cells.CD4.Naive Infiltration Score
CCDC6 expression log2(TPM+0.001)

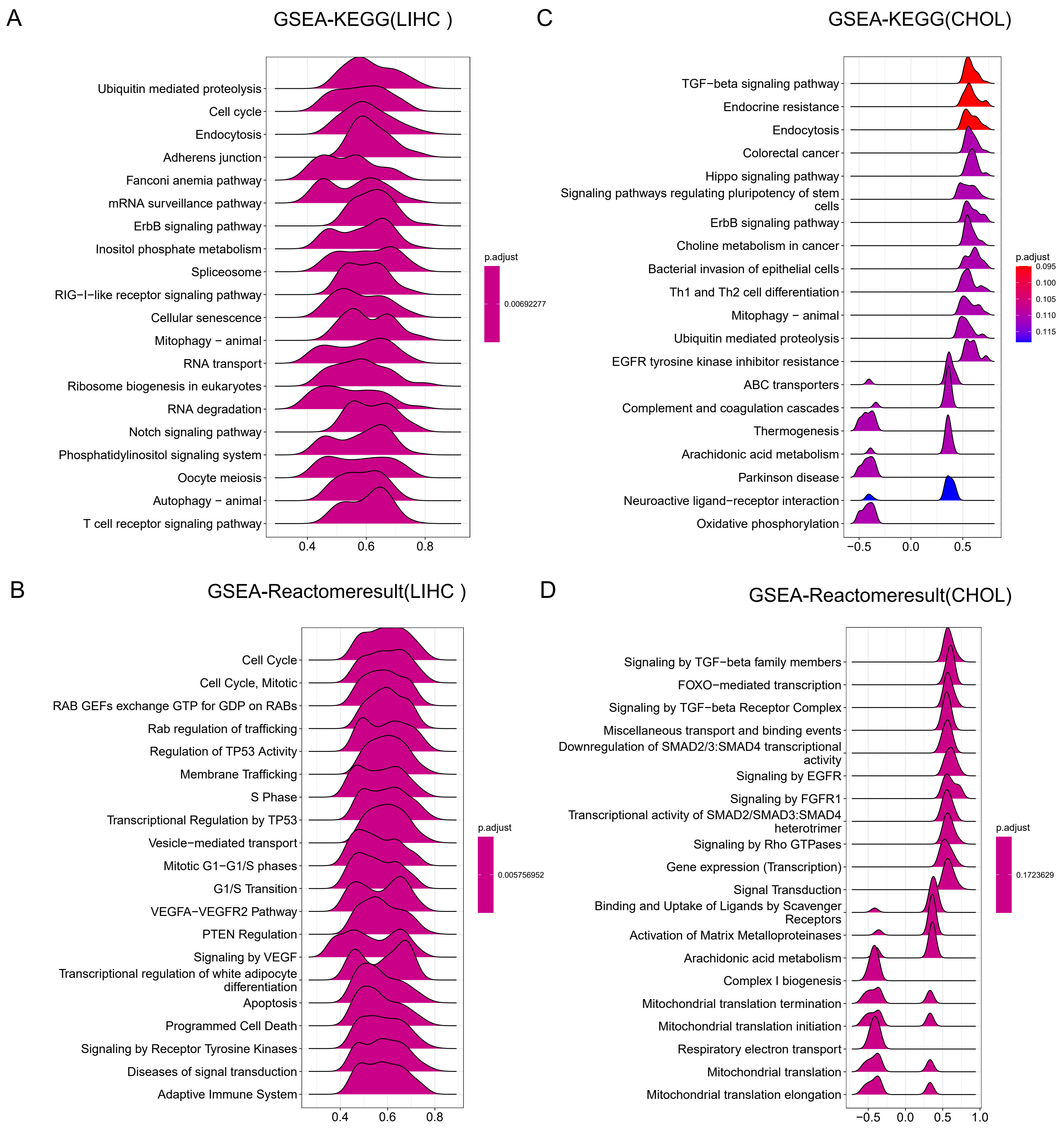
A
GSEA-KEGG(LIHC)
Ubiquitin mediated proteolysis
Cell cycle
Endocytosis
Adherens junction
Fanconi anemia pathway
mRNA surveillance pathway
ErbB signaling pathway
Inositol phosphate metabolism
Spliceosome
RIG−I−like receptor signaling pathway
Cellular senescence
Mitophagy − animal
RNA transport
Ribosome biogenesis in eukaryotes
RNA degradation
Notch signaling pathway
Phosphatidylinositol signaling system
Oocyte meiosis
Autophagy − animal
T cell receptor signaling pathway
0.4
0.6
0.8
p.adjust
0.00692277
C
GSEA-KEGG(CHOL)
TGF−beta signaling pathway
Endocrine resistance
Endocytosis
Colorectal cancer
Hippo signaling pathway
Signaling pathways regulating pluripotency of stem cells
ErbB signaling pathway
Choline metabolism in cancer
Bacterial invasion of epithelial cells
Th1 and Th2 cell differentiation
Mitophagy − animal
Ubiquitin mediated proteolysis
EGFR tyrosine kinase inhibitor resistance
ABC transporters
Complement and coagulation cascades
Thermogenesis
Arachidonic acid metabolism
Parkinson disease
Neuroactive ligand−receptor interaction
Oxidative phosphorylation
−0.5
0.0
0.5
p.adjust
0.095
0.100
0.105
0.110
0.115
B
GSEA-Reactomeresult(LIHC)
Cell Cycle
Cell Cycle, Mitotic
RAB GEFs exchange GTP for GDP on RABs
Rab regulation of trafficking
Regulation of TP53 Activity
Membrane Trafficking
S Phase
Transcriptional Regulation by TP53
Vesicle−mediated transport
Mitotic G1−G1/S phases
G1/S Transition
VEGFA−VEGFR2 Pathway
PTEN Regulation
Signaling by VEGF
Transcriptional regulation of white adipocyte differentiation
Apoptosis
Programmed Cell Death
Signaling by Receptor Tyrosine Kinases
Diseases of signal transduction
Adaptive Immune System
0.4
0.6
0.8
p.adjust
0.005756952
D
GSEA-Reactomeresult(CHOL)
Signaling by TGF−beta family members
FOXO−mediated transcription
Signaling by TGF−beta Receptor Complex
Miscellaneous transport and binding events
Downregulation of SMAD2/3:SMAD4 transcriptional activity
Signaling by EGFR
Signaling by FGFR1
Transcriptional activity of SMAD2/SMAD3:SMAD4 heterotrimer
Signaling by Rho GTPases
Gene expression (Transcription)
Signal Transduction
Binding and Uptake of Ligands by Scavenger Receptors
Activation of Matrix Metalloproteinases
Arachidonic acid metabolism
Complex I biogenesis
Mitochondrial translation termination
Mitochondrial translation initiation
Respiratory electron transport
Mitochondrial translation
Mitochondrial translation elongation
−0.5
0.0
0.5
1.0
p.adjust
0.1723629

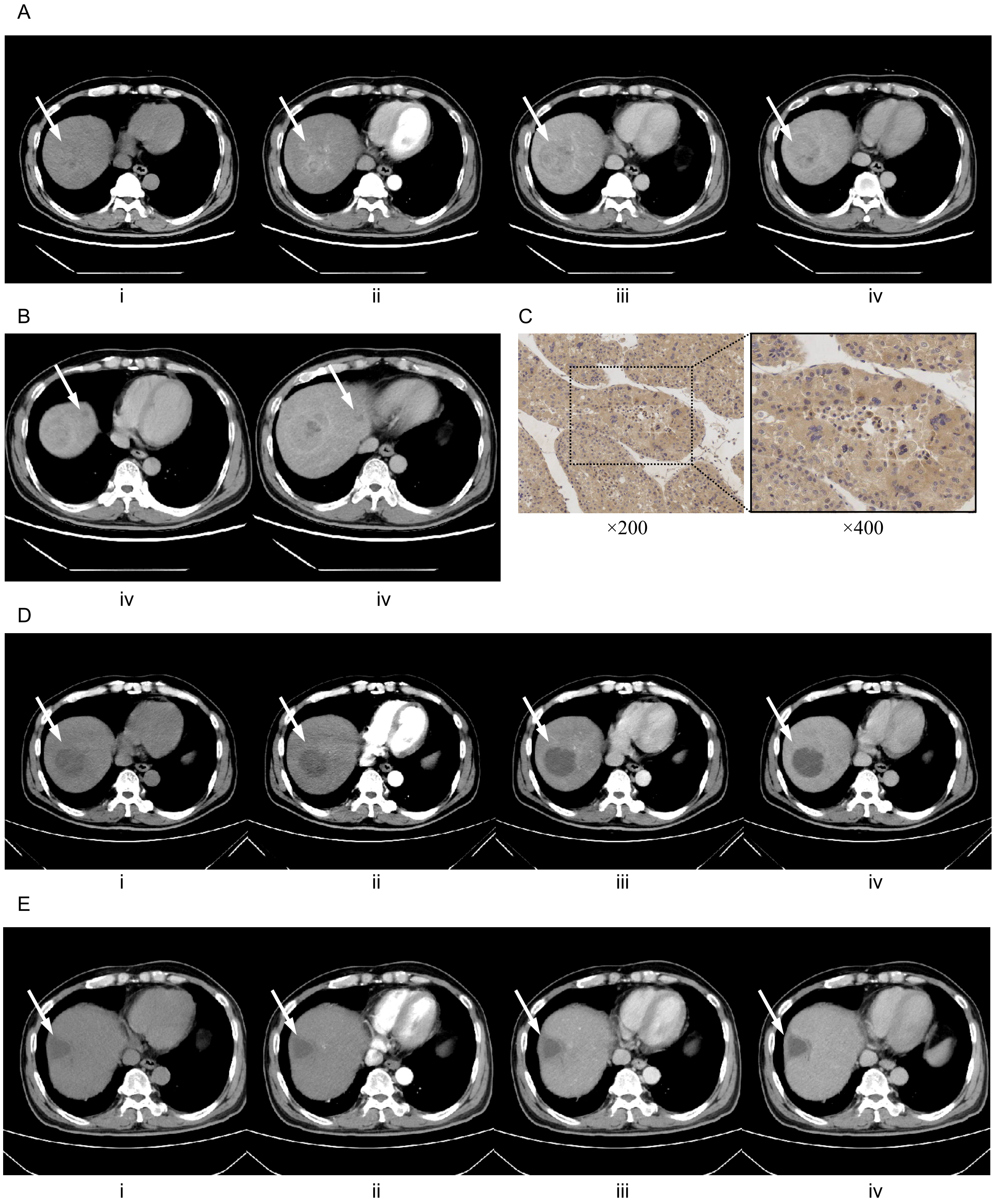
A
i
ii
iii
iv
B
C
iv
iv
×200
×400
D
i
ii
iii
iv
E
i
ii
iii
iv
